# In-situ low-temperature sulfur CVD on metal sulfides with SO_2_ to realize self-sustained adsorption of mercury

**DOI:** 10.1038/s41467-024-47725-3

**Published:** 2024-04-18

**Authors:** Qinyuan Hong, Haomiao Xu, Xiaoming Sun, Jiaxing Li, Wenjun Huang, Zan Qu, Lizhi Zhang, Naiqiang Yan

**Affiliations:** 1 School of Environmental Science and Engineering, Shanghai Jiao Tong University, Shanghai, 200240 China; 2 Shanghai Institute of Pollution Control and Ecological Security, Shanghai, 200092 China; 3 Key Laboratory of Pesticide & Chemical Biology of Ministry of Education Institute of Applied & Environmental Chemistry College of Chemistry, Central China Normal University, Wuhan, 430079 China

**Keywords:** Sustainability, Pollution remediation

## Abstract

Capturing gaseous mercury (Hg^0^) from sulfur dioxide (SO_2_)-containing flue gases remains a common yet persistently challenge. Here we introduce a low-temperature sulfur chemical vapor deposition (S-CVD) technique that effectively converts SO_2_, with intermittently introduced H_2_S, into deposited sulfur (S_d_^0^) on metal sulfides (MS), facilitating self-sustained adsorption of Hg^0^. ZnS, as a representative MS model, undergoes a decrease in the coordination number of Zn–S from 3.9 to 3.5 after S_d_^0^ deposition, accompanied by the generation of unsaturated-coordinated polysulfide species (S_n_^2–^, named S_d_^*^) with significantly enhanced Hg^0^ adsorption performance. Surprisingly, the adsorption product, HgS (ZnS@HgS), can serve as a fresh interface for the activation of S_d_^0^ to S_d_^*^ through the S-CVD method, thereby achieving a self-sustained Hg^0^ adsorption capacity exceeding 300 mg g^−1^ without saturation limitations. Theoretical calculations substantiate the self-sustained adsorption mechanism that S_8_ ring on both ZnS and ZnS@HgS can be activated to chemical bond S_4_ chain, exhibiting a stronger Hg^0^ adsorption energy than pristine ones. Importantly, this S-CVD strategy is applicable to the in-situ activation of synthetic or natural MS containing chalcophile metal elements for Hg^0^ removal and also holds potential applications for various purposes requiring MS adsorbents.

## Abstract (web summary)

Efficient removal of gaseous mercury (Hg^0^) from industrial flue gas is commonly constrained by SO_2_. Here, authors propose an in-situ sulfur CVD method on metal sulfides that reverses the harmful effects of SO_2_, achieving self-sustained adsorption of Hg^0^ and breaking the capacity limitations.

## Introduction

Mercury pollution has aroused global concern mainly ascribed to the volatility, insolubility, and long-range transport of gaseous elemental mercury (Hg^0^), which is the focus of mercury abatement^1–4^. Effective control of Hg^0^ pollutant necessitates the use of functional materials that exhibit high activity, stability, and tolerance to flue gas conditions^5–7^. Recent developments in materials for Hg^0^ capture include carbon-based^8,9^, oxide-based^10^, and noble metal-based materials^11,12^. However, the presence of SO_2_ in any flue gas has been known to negatively impact Hg^0^ removal, leading to surface sulfation or the occupation of active sites^13^. More unfortunately, the non-ferrous metal smelting industry is considered as the largest single source of Hg^0^ emissions, where high concentrations of SO_2_ and Hg^0^ co-exist^14^. Therefore, achieving large-capacity adsorption of Hg^0^ at high concentration of SO_2_ remains a significant challenge.

While many sulfur-based materials have shown a degree of resistance to SO_2_ in Hg^0^ adsorption^15,16^, their capacities often experience significant suppression due to active site depletion or deactivation, particularly at high SO_2_ concentrations^17^. This limitation frequently requires off-line regeneration under harsh conditions (e.g., heating or acidification treatments with irreversible destruction) or even necessitates the replacement of adsorbents^18,19^. To address the challenge of active depletion or deactivation, a more cost-effective and convenient method involves the continuous replenishment of active sites in situ at the interface of sulfur-based materials. Recognizing that the performance of metal sulfides (MS) relies heavily on the quantity of active sulfur sites^20^, a viable strategy is to directly convert SO_2_ to sustainably replenish surface active sites, thereby turning the negative effect of SO_2_ into a positive one. However, the high average S−O bond energy of SO_2_ (548 kJ mol^−1^) necessitates high-temperature conditions (>2000 °C) for its decomposition^21,22^, whereas the preferential reaction of SO_2_ with flue gas O_2_ impedes the feasibility of this pathway^23^. Notably, the assistance of H_2_S can lower the S−O bond breaking energy barrier (139 kJ mol^−1^) and further reorganize the S−S bond to generate elemental sulfur through the Claus reaction^24,25^. Fortunately, H_2_S or its raw materials (Na_2_S or NaHS) are easily accessible and commonly used for heavy metals removal from various wastewaters in non-ferrous smelters^26,27^.

However, two key challenges persist in achieving our objectives. Firstly, it involves effectively generating fresh sulfur on MS surface. More importantly, it pertains to activating the deposited sulfur (S_d_^0^) for site replenishment instead of allowing it to aggregate into the inert S_8_^0^ state (octatomic ring structure with poor Hg^0^ adsorption activity^28,29^). To address these challenges, we have developed a sulfur chemical vapor deposition (S-CVD) method using the Claus reaction between excessive SO_2_ in flue gases and intermittently added H_2_S. This method facilitates the deposition of gas-phase sulfur species with high controllability and scalability. Furthermore, an active interface is crucial to bonding with S_d_^0^ to create unsaturated coordination sites rather than coordination-saturated S_8_. Notably, the incorporation of anchoring sites to bond with sulfur can maintain its unsaturated state^30^. Chalcophile elements exhibit a natural tendency to lose outer electrons to form an 18-electron outermost structure (s^2^p^6^d^10^), which in turn combines with sulfur (3s^2^3p^4^) to form an ionic compound under ambient conditons^31,32^. Thus, MS containing chalcophile metals emerges as promising candidates to bond with S_d_^0^, preventing it from falling into a saturated-coordinated ring structure.

Hence, this work employs the proposed in-situ S-CVD method on chalcophile MS to counteract the negative effects of SO_2_ and achieve self-sustained Hg^0^ adsorption, enabling in-situ reactivation without the need to replace spent adsorbents. Variety of experimental conditions and characterization methods, such as scanning electron microscopy (SEM), X-ray absorption fine structure (XAFS), and density functional theory (DFT) calculations, were devoted to evaluating the self-sustained adsorption performance, revealing the deposition process of S_d_^0^, identifying the formation of unsaturated coordination environments, and calculating the energy changes to interpret the self-sustained adsorption mechanism. The results indicate that S_d_^0^ can be efficiently activated to polysulfide (S_n_^2−^, named S_d_^*^) species by chalcophile MS, including the formed HgS itself, ensuring self-sustained Hg^0^ adsorption. This in-situ S-CVD approach provides a promising solution to active site depletion and poisoning issues and offers an avenue for efficient and continuous heavy metal removal using MS materials.

## Results

### Establishment of in-situ S-CVD method for SO_2_ deposition

**Fig. 1:**
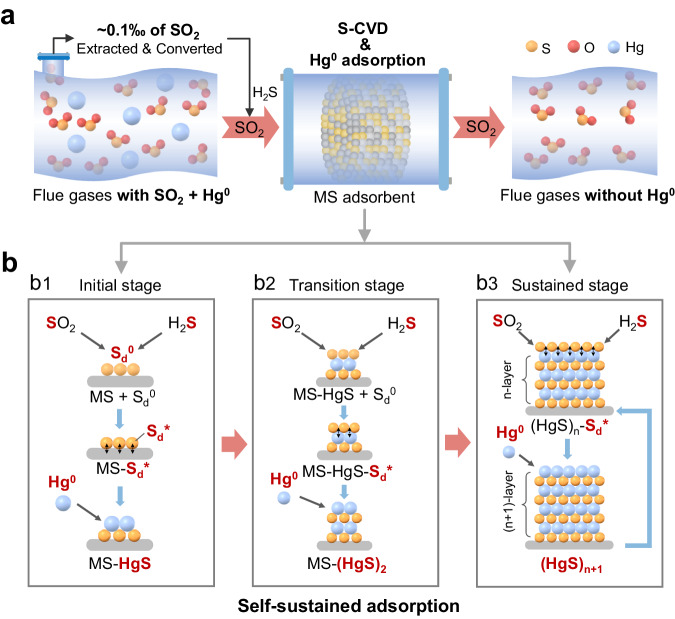
Proposed in-situ S-CVD strategy for Hg^0^ self-sustained adsorption on metal sulfides. **a** Hg^0^ removal through proposed in-situ S-CVD strategy in smelting flue gas. **b** Schematic illustration of the Hg^0^ self-sustained adsorption on metal sulfides. **b1** Initial stage, S_d_^0^ activated only by MS; **b2** Transition stage, S_d_^0^ activated by MS and/or HgS; **b3** Sustained stage, S_d_^0^ activated by HgS itself.

The in-situ S-CVD method was established for flue gas SO_2_ deposition. To initiate S-CVD, a small amount of H_2_S (100 ppm) was injected into the SO_2_-containing flue gas upstream of Al_2_O_3_@MS adsorbents (Supplementary Fig. 1). In actual non-ferrous smelting processes, flue gas particle-bond mercury (Hg^p^) and oxidized mercury (Hg^2+^) can be respectively removed by an electrical precipitator and scrubber, resulting in a subsequent flue gas with high concentrations of SO_2_ and Hg^0^ (Supplementary Fig. 2). Extraction of approximately 0.1‰ of total SO_2_ for on-site conversion to H_2_S^27,33^ can satisfy the needs of S-CVD, and Hg^0^ will be removed by the proposed self-sustained adsorption method on adsorbents (Fig. 1a). In this method, MS functions as the fresh surface for the initial S-CVD and Hg^0^ adsorption, and then the spent MS (i.e., MS-HgS) acts as new surface for further S-CVD and Hg^0^ adsorption, ultimately achieving the sustained adsorption by HgS itself (Fig. 1b).

During the S-CVD process, the reaction ratio of H_2_S and SO_2_ was monitored as 2.1: 1 and chemical composition analysis exhibited that the sulfur content in different Al_2_O_3_@MS increased by 2.1%−3.0% after 180 min of S-CVD (Supplementary Fig. 3). These confirm the occurrence of the Claus reaction on the Al_2_O_3_@MS surface, which is a critical step in the S-CVD process. Furthermore, the optimal adding sequence of H_2_S and SO_2_ was investigated to understand the generation mechanism of S_d_^0^. The results demonstrated that the activity of Al_2_O_3_@MS pretreated with H_2_S followed by SO_2_ was significantly lower than that pretreated with SO_2_ followed by H_2_S (Supplementary Fig. 4). Meanwhile, pretreatment only by SO_2_ cannot enhance the activity of Al_2_O_3_@MS. This finding indicates that the formation of S_d_^0^ on Al_2_O_3_@MS surface followed the Eley-Rideal mechanism, in which SO_2_ is first adsorbed on adsorbent surface and then reacts with gaseous H_2_S to produce S_d_^0^ (Eq. 1, 2):

1 $${{{{{{\rm{SO}}}}}}}_{2}({{{{{\rm{g}}}}}})+{{{{{{\rm{Al}}}}}}}_{2}{{{{{{\rm{O}}}}}}}_{3}{{{{{\rm{@MS}}}}}} \to {{{{{{\rm{Al}}}}}}}_{2}{{{{{{\rm{O}}}}}}}_{3}{{{{{\rm{@MS}}}}}}{\mbox{-}}{{{{{{\rm{SO}}}}}}}_{2}({{{{{\rm{ads}}}}}})$$

2 $${{{{{{\rm{Al}}}}}}}_{2}{{{{{{\rm{O}}}}}}}_{3}{{{{{\rm{@MS}}}}}}{\mbox{-}}{{{{{{\rm{SO}}}}}}}_{2}({{{{{\rm{ads}}}}}})+{2{{{{{\rm{H}}}}}}}_{2}{{{{{\rm{S}}}}}}({{{{{\rm{g}}}}}})\to {{{{{{\rm{Al}}}}}}}_{2}{{{{{{\rm{O}}}}}}}_{3}{{{{{\rm{@MS}}}}}}{\mbox{-}}{3{{{{{\rm{S}}}_{{{\rm{d}}}}^0}}}}+{2{{{{{\rm{H}}}}}}}_{2}{{{{{\rm{O}}}}}}$$

Importantly, the negative Gibbs free energy (Δ*G*^0^ = −91 kJ mol^−1^ at 25 °C) of the Claus reaction and the much lower concentration of H_2_S (100 ppm) compared to SO_2_ (≥5000 ppm) used in S-CVD guarantee the sufficient reaction of added H_2_S.

### Deposited sulfur activation on MS for Hg^0^ removal

**Fig. 2:**
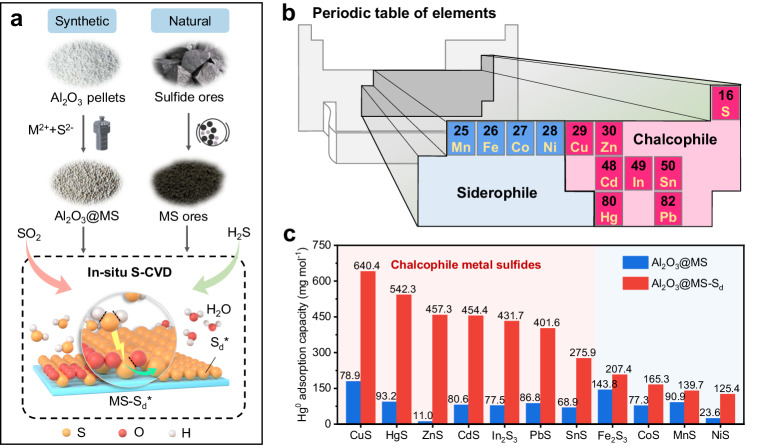
Enhanced performance of different metal sulfides using in-situ SCVD activation. **a** Proposed in-situ S-CVD strategy on synthesized metal sulfides or natural sulfide ores. **b** Geochemical classification of elements containing chalcophile and siderophile elements. **c** Hg^0^ adsorption capacities of different Al_2_O_3_@MS and Al_2_O_3_@MS-S_d_ (M = Cu, Hg, Zn, Cd, In, Pb, Sn, Ni, Co, Fe, and Mn). Reaction conditions: adsorbent mass = 0.3 g, total flow rate = 360 mL min^−1^, SO_2_ concentration = 5000 ppm (during S-CVD process), H_2_S concentration = 100 ppm (during S-CVD process), S-CVD time = 15 min, Hg^0^ concentration = (1.5 ± 0.05) mg m^−3^, reaction temperature = 80 °C, and reaction time = 180 min.

The surface properties of metal sulfides play a vital role in S-CVD process. Both synthetic and natural MS served as the deposition surface (Fig. 2a). In light of Goldschmidt geochemical classification of the elements, metals with chalcophile nature have higher affinity towards sulfur, which could accelerate the stimulation of S_d_^0^ (refs. ^31,32^). Thus, a range of typical chalcophile metal elements, including Cu, Zn, In, Cd, Pb, and Sn, were chosen as candidates for the synthesis of MS, while some siderophile metals, including Mn, Fe, Co, and Ni, were offered as contrasts (Fig. 2b). As depicted in Fig. 2c and Supplementary Table 1, after 15 min of S-CVD, various Al_2_O_3_@MS-S_d_ showed substantial differences in enhancing their Hg^0^ adsorption performances, of which all the chalcophile Al_2_O_3_@MS-S_d_ exhibited significantly increase in their adsorption capacities. Notably, Al_2_O_3_@CuS-S_d_ demonstrated a remarkable increase the Hg^0^ adsorption capacity, rising from 178.9 to 640.4 mg mol^−1^(within 180 min and normalized to MS molar mass). Similarly, Al_2_O_3_@ZnS-S_d_ exhibited a significant enhancement, with adsorption capacity increasing from 11.0 to 457.3 mg mol^−1^. Al_2_O_3_@CdS-S_d_, Al_2_O_3_@In_2_S_3_-S_d_, Al_2_O_3_@PbS-S_d_, and Al_2_O_3_@SnS-S_d_ also showed considerable improvements in their Hg^0^ adsorption capacities, reaching 454.4, 431.7, 401.6, and 275.9 mg mol^−1^, respectively. However, the adsorption capacities of Al_2_O_3_@Fe_2_S_3_-S_d_, Al_2_O_3_@CoS-S_d_, Al_2_O_3_@MnS-S_d_, and Al_2_O_3_@NiS-S_d_ were comparatively lower, reaching 207.4, 165.3, 139.7, and 125.4 mg mol^−1^, respectively. In addition, the Al_2_O_3_-S_d_, Al_2_O_3_@ZnSO_4_-S_d_, and Al_2_O_3_@Na_2_S-S_d_ did not show improved performance compared to their raw materials (Supplementary Fig. 5), implying that the isolated presence of metal or sulfur sites cannot directly activate the deposited S_d_^0^.

Further, to quantitatively explore the role of metal sites in MS on the activation of S_d_^0^, we further construct the relationship between the Hg^0^ adsorption capacity increment (*Q*_i_ = *Q*_Al2O3@MS-Sd_ − Q_Al2O3@MS_) and metal-sulfur (M−S) bond energy (measured by bond length^34^, Supplementary Table 2) of the investigated metal sulfides. As depicted in Supplementary Fig. 6, the Q_i_ of chalcophile MS showed a negative relationship with the increase of M−S bond length. However, for siderophile MS, higher M−S bond length instead led to relatively higher Q_i_. It can be deduced that the activity of S_d_^0^ on the sulfide interface highly depends on the geochemical characteristics of metal element and the M−S affinity. Notably, Hg also belongs to chalcophile elements (Fig. 2b), and it is supposed that HgS itself could potentially play a role in activating S_d_^0^ (see later in Fig. 5e).

**Fig. 3:**
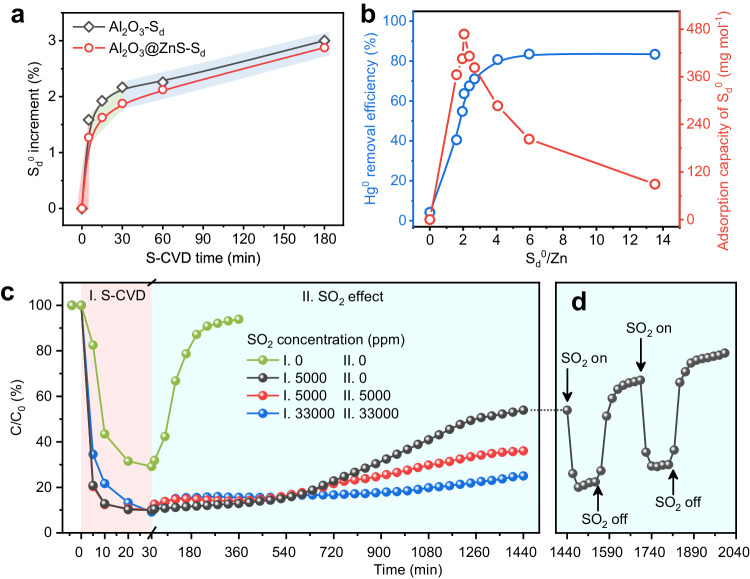
Influencing factors of in-situ SCVD strategy for Hg^0^ adsorption over Al_2_O_3_@ZnS-S_d_. **a** S_d_^0^ increment in Al_2_O_3_ and Al_2_O_3_@ZnS with the increase of S-CVD time. The red, green and blue-gray highlights represent three stages of different S_d_^0^ growth rates. **b** The relationship between S_d_^0^ increment and Hg^0^ adsorption capacity of Al_2_O_3_@ZnS-S_d_. **c** The effect of SO_2_ concentration on Hg^0^ adsorption breakthrough curve of Al_2_O_3_@ZnS-S_d_. **d** The effect of intermittent addition of SO_2_ on the Hg^0^ adsorption curve over Al_2_O_3_@ZnS-S_d_. Reaction conditions: adsorbent mass = 0.3 g, total flow rate = 360 mL min^−1^, Hg^0^ concentration = (1.5 ± 0.05) mg m^−3^, SO_2_ concentration = 5000 ppm (for **a** and **b**), H_2_S concentration = 100 ppm (during the S-CVD process), temperature = 80 °C (for **a**), and S-CVD time = 30 min (for **b** and **c**).

We then choose ZnS as a representative model to optimize the reaction conditions owing to its significant performance enhancement (~42 times) and simple metal and sulfur speciation. The sulfur content in Al_2_O_3_@ZnS-S_d_ exhibited a three-stage growth pattern with increasing S-CVD time (Fig. 3a). The growth rate of S_d_^0^ decreased from 2.5 mg g^−1^ min^−1^ at 0−5 min to 0.2 mg g^−1^ min^−1^ at 5−30 min then to 0.06 mg g^−1^ min^−1^ at >30 min (Supplementary Table 3). The decreasing formation rate indicates that the fresh surface of the adsorbent was gradually covered by S_d_^0^, and once the surface was completely covered, subsequent S_d_^0^ would generate on the existing S_d_^0^ layer, resulting in a final slow but steady growth rate. Figure 3b presents the relationship between S_d_^0^ increment and Hg^0^ removal efficiency of Al_2_O_3_@ZnS-S_d_. The results showed that the removal efficiency gradually increased to 71.7% when the ratio of S_d_^0^/Zn increased to 2.7 (corresponding to 60 min of S-CVD); while, as the ratio further increased to 4.1 (240 min), 6.0 (480 min), and 13.5 (1440 min), the removal efficiency remained at a stable level of around 83%. This indicates that excess deposited S_d_^0^ did not work, presumably attributed to the inevitable aggregation of excess S_d_^0^ into inert S_8_^0^ (ref. ^29^). When the adsorption capacity is normalized to the mole of S_d_^0^ (red curve in Fig. 3b), it reached a maximum of 467.5 mg mol^−1^ at the S_d_^0^/Zn ratio of 2.1 (15 min) and then gradually decreased to 89.3 mg mol^−1^ as the ratio increased to 13.5. Therefore, 30 min of S-CVD was chosen as the optimal condition by integrating the Hg^0^ removal efficiency and S_d_^0^ utilization. Besides S-CVD time, reaction temperatures and other flue gas components also played significant roles. Thermogravimetric analysis (TGA) results indicated the high thermal stability of Al_2_O_3_@ZnS-S_d_ below 200 °C (Supplementary Fig. 7). The increase in adsorption temperature from 60 to 120 °C improved the Hg^0^ removal efficiency of Al_2_O_3_@ZnS-S_d_ from 23.8% to 89.9% within 180 min (Supplementary Fig. 8). However, further elevation of temperature to 140 °C and 160 °C resulted in decreasing activity to 86.6% and 75.9%, respectively, presumably due to the re-decomposition of partially captured Hg^0^ (Supplementary Fig. 9). Moreover, Al_2_O_3_@ZnS-S_d_ demonstrated high tolerance to different gas components (Supplementary Fig. 10). The addition of 5000 ppm SO_2_ in adsorption process resulted in enhanced removal efficiency by 3.2% at 120 °C, and further addition of 5% O_2_ or 100 ppm NO had slight influence with a reduction of 2.6% and 6.8%, respectively. The introduction of 5000 ppm SO_2_ + 4% H_2_O showed negative effect on Hg^0^ adsorption performance of Al_2_O_3_@ZnS-S_d_, presumably due to the competing adsorption between H_2_O and Hg^0^ on the active sites^35^ and the hydrophilicity of Al_2_O_3_ (ref. ^36^); however, its removal efficiency maintained at a stable level of ~70% without reduction for 180 min adsorption.

Long-term experiments were conducted to clarify the SO_2_ effect on Hg^0^ removal during the S-CVD and adsorption process under the optimal condition (H_2_S = 100 ppm, temperature = 120 °C, S-CVD time = 30 min) (Fig. 3c). In stage I (S-CVD process), without the presence of SO_2_, Al_2_O_3_@ZnS initially exhibited a temporary increase in Hg^0^ removal but rapidly decreased once the S-CVD was stopped. This improvement might be attributed to the sulfuration of residual metal salt precursors on the surface. However, when SO_2_ was added during the S-CVD process, the Hg^0^ removal sharply increased to around 90%. Furthermore, SO_2_ continued to exhibit an extraordinary effect on the performance of Al_2_O_3_@ZnS-S_d_ after the S-CVD process. As shown in stage II (without H_2_S), the slope of curve without SO_2_ addition in the adsorption process (black line) was lower than that with SO_2_ addition (red one), indicating a positive effect of SO_2_, which is contrary to our traditional perceptions. Moreover, as the SO_2_ concentration increased from 0 ppm to 5000 ppm and to 33,000 ppm, the Hg^0^ removal after 1440 min reaction increased from 46.1% to 64.0% and to 75.0%, respectively. To verify this unexpected positive effect, SO_2_ was added intermittently to the SO_2_-free system in stage II (Fig. 3d). The results showed that once 5000 ppm of SO_2_ was introduced, the Hg^0^ removal efficiency was instantly improved by about 35%; however, once the SO_2_ was turned off, it dropped back down to a lower level immediately. Thus, SO_2_ was identified as the positive component for Hg^0^ adsorption over the Al_2_O_3_@ZnS-S_d_. Through Hg^0^ temperature-programmed desorption (Hg^0^-TPD) experiments, we analyzed the changes in de-mercury products (Supplementary Fig. 9). The desorption peaks of spent Al_2_O_3_@ZnS-S_d_ in the absence of SO_2_ located at 210−265 °C, which are attributed to the decomposition temperature of β-HgS^37^. While, after adsorption in the presence of SO_2_, there appeared a new peak centered at higher temperature of 365 °C, which is assigned to the decomposition temperature of α-HgS^38^, thereby improving its Hg^0^ adsorption stability. Additionally, the performance of Al_2_O_3_@MS without S-CVD assistance was significantly reduced at high concentrations of SO_2_, excluding the promotional effect of SO_2_ on Al_2_O_3_@MS itself (Supplementary Fig. 11).

### Self-sustained adsorption performance of Al_2_O_3_@MS-S_d_

**Fig. 4:**
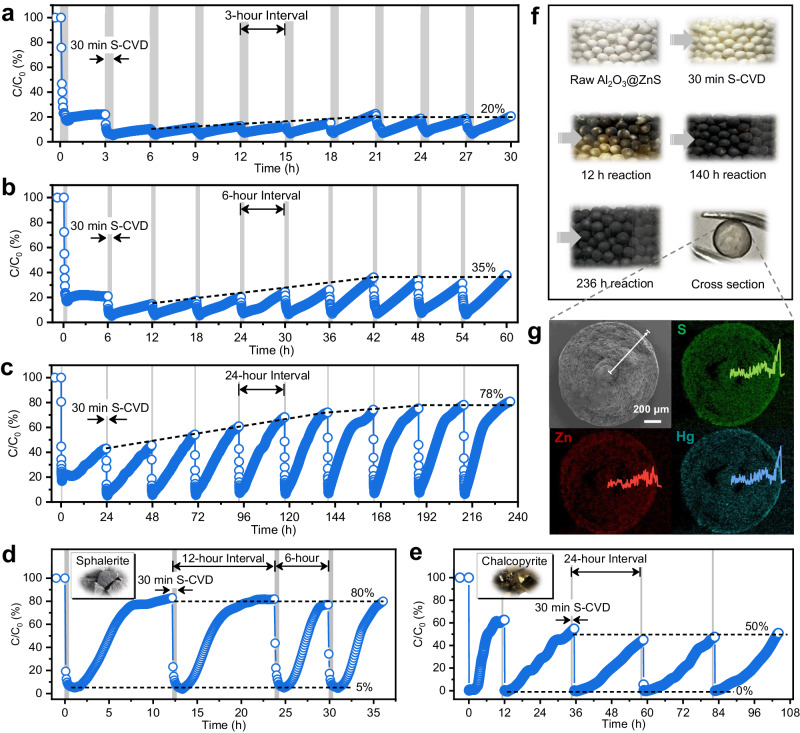
Self-sustained adsorption performance of Hg^0^. Hg^0^ adsorption breakthrough curves of Al_2_O_3_@ZnS-S_d_ assisted with (**a**) 3 h, **b** 6 h, and **c** 24 h intermittent S-CVD. Hg^0^ adsorption breakthrough curves of (**d**) natural chalcopyrite ore and (**e**) sphalerite ore assisted with intermittent S-CVD. Insets in **d** and **e**: photographs of the corresponding natural ores. Reaction conditions: adsorbent mass = 0.4 g (for **a**–**c**) or 1 g (for **d** and **e**), temperature = 120 °C (for **a**–**d**) or 40 °C (for **e**), [Hg^0^] = (2.5 ± 0.05) mg m^−3^, [SO_2_] = 5000 ppm (for **a**–**c**) or 6% (for **d** and **e**), [H_2_O] = 4%, [H_2_S] = 100 ppm (during S-CVD), and total flow rate = 300 mL min^−1^. **f** Photographs of Al_2_O_3_@ZnS in different reaction stages. **g** EDS mapping images of the cross-section of spent Al_2_O_3_@ZnS-S_d_. Inset: line scanning results of the selected position.

Although Al_2_O_3_@ZnS-S_d_ exhibited high performance and SO_2_ can further promote the Hg^0^ adsorption, it still has a limited number of active sites and requires replacement of the adsorbent once depleted. If we can periodically replenish the S_d_^0^ on the spent Al_2_O_3_@ZnS-S_d_ surface, self-sustained adsorption of Hg^0^ can be achieved without the need for adsorbent replacement. Hence, simulated flue gas ((2.5 ± 0.05) mg m^−3^ Hg^0^, 5000 ppm SO_2_, 4% H_2_O, total flow rate = 300 mL min^−1^, and reaction temperature = 120 °C) was applied to investigate the self-sustained adsorption performance of Al_2_O_3_@ZnS-S_d_. 100 ppm of H_2_S was intermittently injected into the flue gas for 30 min per 3, 6, and 24 h to replenish S_d_^0^ (Fig. 4a–c). The Hg^0^ removal of Al_2_O_3_@ZnS-S_d_ can reach around 95% after each round of S-CVD except the first round (83.2%). After the supplemented S_d_^0^ was consumed by Hg^0^, the removal efficiency gradually decreased. Meanwhile, as the number of S-CVD increased, the 3, 6, and 24 h breakthrough ratios gradually converged to ~20%, ~35%, and ~78%, respectively. Additionally, owing to the higher performance of Al_2_O_3_@CuS-S_d_ at low temperatures (Supplementary Fig. 12), it displayed the self-sustained adsorption performance at 60 °C, with an initial Hg^0^ removal efficiency of ~90% and a 24 h breakthrough ratio of ~50% in each round of reaction (Supplementary Figs. 13). Moreover, Al_2_O_3_@ZnS-S_d_ can restrore its original activity after Hg^0^ desorption and secondary S-CVD (Supplementary Fig. 14). Additionally, we evaluated the Hg^0^ re-emission of Al_2_O_3_@ZnS-S_d_ after ten rounds of reaction. At the reaction temperature, the Hg^0^ re-emission concentration can be reduced from 1.7 mg m^−3^ to 0.05 mg m^−3^ (emission standard for non-ferrous smelting flue gas in China) in 30 min with the assistance of S-CVD, and once the temperature dropped to room temperature, the Hg^0^ concentration rapidly decreased to 0 mg m^−3^ (Supplementary Fig. 15). Thus, the S-CVD strategy not only can fulfill the self-sustained adsorption of Hg^0^, but also inhibit the re-emission of adsorbed mercury.

Besides synthesized Al_2_O_3_@MS, natural sulfide ores that contain chalcophile metal elements, such as chalcopyrite and sphalerite, also enable their potential for self-sustained Hg^0^ adsorption. As depicted in Fig. 4d, at conditions of near-actual flue gas SO_2_ concentration (6%), natural sphalerite ore achieved ~95% Hg^0^ removal after each round of S-CVD, and the breakthrough ratio converged to ~80%. Chalcopyrite exhibited enhanced Hg^0^ adsorption performance with intermittent S-CVD at a lower temperature (40 °C), which reached a 100% initial Hg^0^ removal efficiency and had a 24 h breakthrough ratio of ~50% in each round of reaction (Fig. 4e). Thus, directly utilization of natural sulfide ores as adsorbents can effectively lower the cost for Hg^0^ pollution control as well as improve the adsorption capacity taking advantage of their self-sustained adsorption properties.

Figure 4f shows the macrophotographs of Al_2_O_3_@ZnS in different reaction stages. After the first round of S-CVD, the color of Al_2_O_3_@ZnS changed from white to pale yellow, verifying the formation of S_d_^0^. Then, the color of spent Al_2_O_3_@ZnS-S_d_ gradually turned black after 140 h reaction (six rounds of S-CVD and Hg^0^ adsorption). The cross-sectional view of spent Al_2_O_3_@ZnS-S_d_ pellets (after ten rounds) in Fig. 4f depicted an obvious black shell. The energy dispersion X-ray spectroscopy (EDS) mapping images and the selected line scanning curves of the cross-section of Al_2_O_3_@ZnS revealed that the contents of S and Zn elements on Al_2_O_3_ pellet gradually decreased from outside in (Supplementary Fig. 16). After reaction, S and Hg elements were more scattered in the outer layer and exhibited synchronous linear increase at the boundary layer (~50 μm); while, there presented a decline in Zn element at this boundary layer (Fig. 4g). In addition, the S and Hg contents on the surface of the spent Al_2_O_3_@ZnS-S_d_ was much higher than the Zn content (Supplementary Fig. 17). This indicates a layer-by-layer outward deposition of S_d_^0^ and adsorption of Hg^0^ on Al_2_O_3_@ZnS surface. Moreover, assume that the Zn: S ratio in raw Al_2_O_3_@ZnS is 1:1, the ratio of Hg to S_d_^0^ in spent Al_2_O_3_@ZnS-S_d_ was calculated as 1.04 in light of the ESD result of the cross-section (Supplementary Fig. 18), giving an indication that S_d_^0^ atoms were fully utilized for Hg^0^ adsorption.

### Mechanism for self-sustained Hg^0^ adsorption on Al_2_O_3_@MS-S_d_

**Fig. 5:**
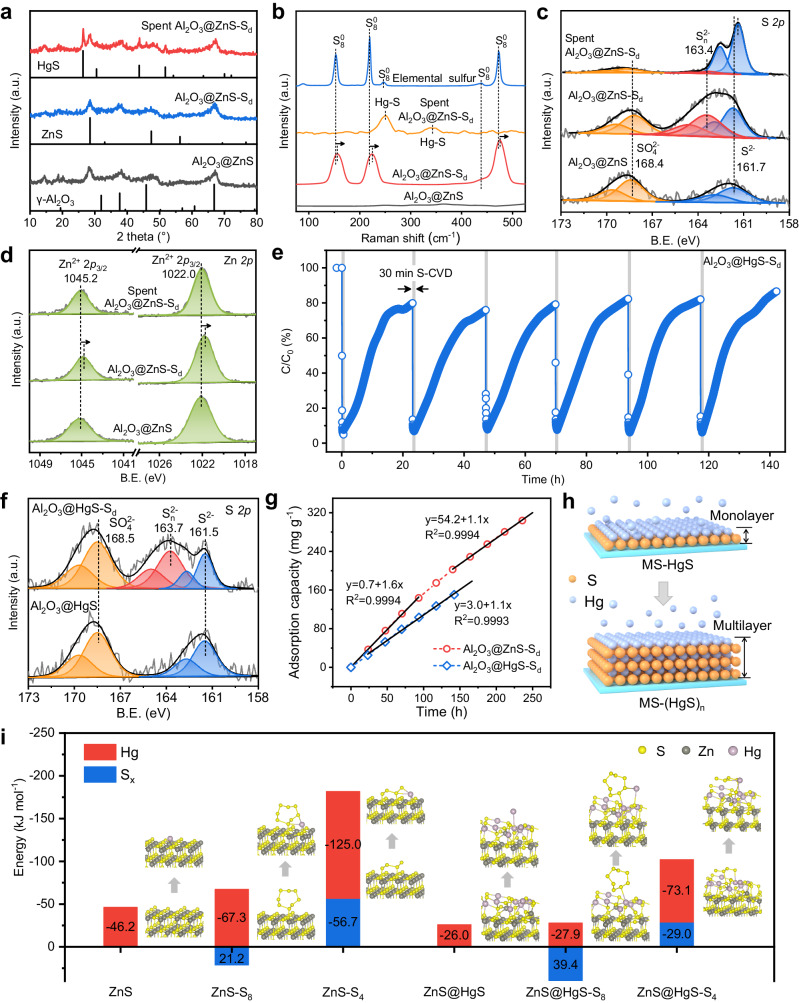
Self-sustained adsorption mechanism. **a** XRD patterns of Al_2_O_3_@ZnS, Al_2_O_3_@ZnS-S_d_, and spent Al_2_O_3_@ZnS-S_d_. **b** Raman spectra of Al_2_O_3_@ZnS, Al_2_O_3_@ZnS-S_d_, spent Al_2_O_3_@ZnS-S_d_, and pure S_8_. **c** S 2*p* and **d** Zn 2*p* XPS spectra of Al_2_O_3_@ZnS, Al_2_O_3_@ZnS-S_d_, and spent Al_2_O_3_@ZnS-S_d_. **e** Hg^0^ adsorption breakthrough curve of Al_2_O_3_@HgS-S_d_ assisted with intermittent S-CVD. Reaction conditions: adsorbent mass = 0.4 g, [Hg^0^] = (2.5 ± 0.05) mg m^−3^, [SO_2_] = 5000 ppm, [H_2_O] = 4%, [H_2_S] = 100 ppm (30 min per 24 h), and total flow rate = 300 mL min^−1^. **f** S 2*p* XPS spectra of Al_2_O_3_@HgS and Al_2_O_3_@HgS-S_d_. **g** Linear fitting of the adsorption rates of Al_2_O_3_@ZnS-S_d_ and Al_2_O_3_@HgS-S_d_ at 120 °C. **h** Schematic illustration of in-situ intermittent S-CVD on Al_2_O_3_@MS surface for Hg^0^ multilayer adsorption. **i** DFT calculations of S_d_^0^ activation and Hg^0^ self-sustained adsorption behaviors on ZnS (111) surface.

Identifying the S_d_^0^ activation and Hg^0^ adsorption behaviors on Al_2_O_3_@ZnS contributes to a profound insight into the self-sustained adsorption mechanism of Hg^0^. The Brunauer−Emmett−Teller (BET) surface area, total pore volume, and average pore size of Al_2_O_3_, Al_2_O_3_@ZnS, and Al_2_O_3_@ZnS-S_d_ were not significantly different (Supplementary Table 4), suggesting that the coating of ZnS and the deposition of S_d_^0^ did not affect the pore structure of Al_2_O_3_. The X-ray diffraction (XRD) pattern of Al_2_O_3_@ZnS-S_d_ showed that, besides the diffraction peaks assigned to γ-Al_2_O_3_ (JCPDS no. 79-1558) and sphalerite ZnS (JCPDS no. 77-2100), no peaks related to elemental sulfur emerged (Fig. 5a), suggesting the amorphous structure of formed S_d_^0^. The Raman spectrum of Al_2_O_3_@ZnS-S_d_ charactered the peaks at 155.6, 223.9, 443.8, and 475.3 cm^–1^, which are related higher than Raman shift of S_8_^0^ (Fig. 5b). This indicates that the surface ZnS can change its S–S vibration of S_d_^0^ and prevent its aggregation^39^. In the X-ray photoelectron spectroscopy (XPS) S 2*p* spectrum of Al_2_O_3_@ZnS-S_d_, aside from the peaks ascribed to S^2−^ (161.7 eV and 162.9 eV)^40^ and SO_4_^2−^ (168.4 eV and 169.6 eV)^41^, new peaks related to S_n_^2−^ (163.4 eV and 164.6 eV)^42^ occurred (Fig. 5c). Additionally, the proportion of S_n_^2−^ in Al_2_O_3_@CuS-S_d_ increased from 21.6% to 29.7% (Supplementary Fig. 19, Supplementary Table 5), demonstrating the consistency of the sulfur chemical state on chalcophile metal sulfides. However, in comparison, Al_2_O_3_-S_d_ featured its characteristic peaks of S 2*p*_3/2_ at binding energies of 164.0 eV and 167.8 eV (Supplementary Fig. 20), which were ascribed to S_8_^0^ and adsorbed unreacted SO_2_ species, respectively^43^. Moreover, the binding energy of Zn^2+^ 2*p*_3/2_ in Al_2_O_3_@ZnS-S_d_ shifted from 1022.0 eV to 1021.8 eV after S_d_^0^ deposition (Fig. 5d), indicating the formation of unsaturated coordination environments^44^. To better observe the microscopic changes and the dynamic evolution of deposited S_d_^0^, pure ZnS was further synthesized in the same way without adding Al_2_O_3_ pellets to directly serve as support for S-CVD process. The transmission electron microscope (TEM) images of ZnS-S_d_ showed a decrease in the contrasts of zinc atoms (Supplementary Fig. 21), indicating the formation of Zn defects^19^. The XAFS S L-edge spectra confirmed the formation of S_n_^2−^ species in ZnS-S_d_ (Supplementary Fig. 22a). The extended XAFS (EXAFS) Zn K-edge spectra further revealed a decrease from 3.9 to 3.5 in the coordination number of Zn to S atoms in ZnS-S_d_ compared to that in pristine ZnS (Supplementary Fig. 22b and c, and Supplementary Table 6), further certifying the formation of unsaturated coordination sites. The XPS depth profiling results depicted that with the Ar^+^ etching depth increased to 6 mm, the average valence state of S_n_^2−^ decreased, suggesting a shortening of the S_n_^2−^ chain length close to the ZnS surface (Supplementary Fig. 23). Furthermore, in-situ Raman spectra revealed conversion of S_8_ to S_n_^2−^ in ZnS-S_d_ at elevated temperatures (Supplementary Fig. 24), which is also demonstrated by Fourier transform infrared spectroscopy (FTIR) and ^13^C nuclear magnetic resonance (NMR) using propylene as an indicator (Supplementary Fig. 25). Therefore, these imply that S_d_^0^ does not simply physically accumulate on the Al_2_O_3_@ZnS surface in form of S_8_^0^ (Eq. 3), but can be activated by Zn atoms and generated chemically bonded S_n_^2−^ (S_d_^*^) with unsaturated coordination environments (Eq. 4):

3 $${{{{{{\rm{Al}}}}}}}_{2}{{{{{{\rm{O}}}}}}}_{3}+{{{{{\rm{S}}}_{{{\rm{d}}}}^0}}}\to {{{{{{\rm{Al}}}}}}}_{2}{{{{{{\rm{O}}}}}}}_{3}{\mbox{-}}{{{{{\rm{S}}}_{{{\rm{d}}}}^0}}}\to {{{{{{\rm{Al}}}}}}}_{2}{{{{{{\rm{O}}}}}}}_{3}{\mbox{-}}{{{{{\rm{S}}}_{{{\rm{8}}}}^0}}}$$

4 $${{{{{{\rm{Al}}}}}}}_{2}{{{{{{\rm{O}}}}}}}_{3}{{{{{\rm{@ZnS}}}}}}+{{{{{\rm{S}}}_{{{\rm{d}}}}^0}}}\to {{{{{{\rm{Al}}}}}}}_{2}{{{{{{\rm{O}}}}}}}_{3}{{{{{\rm{@ZnS}}}}}}{\mbox{-}}{{{{{{\rm{S}}}}}}}_{{{{{\rm{d}}}}}}^{\ast }$$

The kinetic simulation revealed that the Hg^0^ adsorption over Al_2_O_3_@ZnS-S_d_ closely followed the pseudo-first-order kinetic model (Supplementary Fig. 26), emphasizing the important role of the external surface area for Hg^0^ adsorption progress. Notably, after Hg^0^ adsorption, there brought out the crystal of β-HgS (JCPDS no. 75-1538), which characterized its main diffraction peaks at 26.4°, 30.6°, 43.8°, and 51.9°, in the XRD pattern of spent Al_2_O_3_@ZnS-S_d_ (Fig. 5a). The Raman spectrum of spent Al_2_O_3_@ZnS-S_d_ also verified the formation of Hg−S bonds that located at 249.6 cm^−1^ and 343.6 cm^−1^ (Fig. 5b)^45^. Besides, the proportion of S_n_^2–^ species in XPS spectra of spent Al_2_O_3_@ZnS-S_d_ decreased from 36.7% to 3.0%, while the proportion of S^2–^ increased from 35.4% to 82.9% (Fig. 5c, Supplementary Table 5). Moreover, the Hg 4f spectrum of spent Al_2_O_3_@ZnS-S_d_ exhibited the characteristic peaks of Hg^2+^ 4*f*_7/2_ and 4*f*_5/2_ centering at 100.3 eV and 104.3 eV, respectively (Supplementary Fig. 27). This signifies that adsorbed Hg^0^ can combine with the active S_d_^*^ on Al_2_O_3_@ZnS-S_d_ to form HgS:

5 $${{{{{{\rm{Al}}}}}}}_{2}{{{{{{\rm{O}}}}}}}_{3}{{{{{\rm{@ZnS}}}}}}{\mbox{-}}{{{{{{\rm{S}}}}}}}_{{{{{\rm{d}}}}}}^{\ast }+{{{{{{\rm{Hg}}}}}}}^{0}\to {{{{{{\rm{Al}}}}}}}_{2}{{{{{{\rm{O}}}}}}}_{3}{{{{{\rm{@ZnS}}}}}}{\mbox{-}}{{{{{\rm{HgS}}}}}}$$

As the 24 h breakthrough rate increased and stabilized with increasing S-CVD rounds, and HgS crystal was observed in the spent Al_2_O_3_@ZnS-S_d_, we suspect that the adsorbent surface was gradually covered by produced HgS, which may also possess the ability to activate S_d_^0^ owing to the chalcophile nature of Hg element^46^. Upon this, Al_2_O_3_@HgS was synthesized and used to testify the performance of Al_2_O_3_@HgS-S_d_ for Hg^0^ adsorption. As shown in Fig. 5e, Al_2_O_3_@HgS-S_d_ exhibited an initial Hg^0^ removal efficiency of ~96% and an 24 h breakthrough ratio of ~80% in each round of experiment, which is close to that of Al_2_O_3_@ZnS-S_d_ at the tenth round. As shown in Fig. 5f, compared with Al_2_O_3_@HgS, the XPS S 2*p* spectrum of Al_2_O_3_@HgS-S_d_ brought out new sulfur species located at 163.7 eV and 164.9 eV, which were between the binding energies of S_n_^2–^ in Al_2_O_3_@ZnS-S_d_ and S_8_^0^ in Al_2_O_3_-S_d_. This indicates that the average chemical valence of S_d_^*^ on Al_2_O_3_@HgS-S_d_ was slightly higher than that on Al_2_O_3_@ZnS-S_d_, explaining the decrease in Hg^0^ adsorption on Al_2_O_3_@ZnS with increasing S-CVD rounds. The Hg 4 *f* spectra showed that the locations of Hg^2+^ in Al_2_O_3_@HgS-S_d_ shifted to lower binding energies compared to those of Al_2_O_3_@HgS (Supplementary Fig. 28). Moreover, the relationship between the Q_i_ of Al_2_O_3_@HgS-S_d_ and the Hg–S bond energy likewise fell within the negative correlation trend of chalcophile MS (Fig. 2c, Supplementary Fig. 29, Supplementary Table 2).

Notably, as the segmented linear fitting results in Fig. 5g present, with the reaction proceeded, the adsorption rate of Al_2_O_3_@ZnS-S_d_ at 120 °C decreased from 1.6 mg g^–1^ h^–1^ to 1.1 mg g^–1^ h^–1^, which converged to that of Al_2_O_3_@HgS-S_d_ (1.1 mg g^–1^ h^–1^). The adsorption rate of Al_2_O_3_@CuS-S_d_ at 60 °C decreased from 1.5 mg g^–1^ h^–1^ to 1.4 mg g^–1^ h^–1^, which was also converged to that of Al_2_O_3_@HgS-S_d_ (1.3 mg g^–1^ h^–1^) (Supplementary Fig. 30). This validates the gradual transition of S_d_^0^ activation and Hg^0^ adsorption from the raw MS surface to the self-sustained HgS surface, in accordance with the concept presented in Fig. 1b. This verifies the promoting effect of MS on S_d_^0^ is gradually replaced by HgS. Importantly, disregarding the effect of increasing adsorbent size caused by the formation of HgS layer, theoretically, the Hg^0^ adsorption capacity of Al_2_O_3_@ZnS-S_d_ can be continuously increased due to the self-sustained adsorption performance of HgS itself (Fig. 5h). Thus, according to the adsorption rate, the adsorption capacity of Al_2_O_3_@ZnS-S_d_ can achieve (54.2 + 25.1d) mg g^−1^ (d > 6 days), thereby breaking the saturation limitations and realizing multilayer adsorption.

DFT calculations were applied to elaborate the Hg^0^ self-sustained adsorption mechanism on ZnS. ZnS (111) model was established and optimized to investigate the Gibbs free energy (Δ*G*) of S_d_^0^ activation and the adsorption energy (*E*_ads_) of Hg^0^. Given that S_n_^2–^ dominated in the Hg^0^ adsorption process, the Δ*G* from S_8_ ring to S_n_ (*n* = 6, 4, 2, and 1) chains were first calculated. As presents in Supplementary Fig. 31, the ZnS-S_8_ can spontaneously convert to ZnS-S_6_ and then to ZnS-S_4_ with a negative Δ*G* of −77.9 kJ mol^−1^, while the conversion of ZnS-S_4_ to ZnS-S_2_ has a positive Δ*G* of 158.6 kJ mol^−1^. This indicates the most stable structure of ZnS-S_4_. Moreover, compared with ZnS, the Zn–S bond length in ZnS-S_4_ surface increased from 2.31 Å to 2.38–2.48 Å (Supplementary Fig. 32), in line with the decrease in Zn–S coordination number in ZnS-S_d_. The *E*_ads_ of Hg^0^ adsorption on ZnS, ZnS-S_8_, and ZnS-S_4_ were calculated as –46.2, –67.6, and –125.0 kJ mol^−1^, respectively (Fig. 5i). The highest *E*_ads_ of ZnS-S_4_ verifies the important role of S_4_ chain on Hg^0^ adsorption. Further, considering the formation of HgS on Al_2_O_3_@ZnS-S_d_ after self-sustaied adsorption of Hg^0^, we constructed the ZnS@HgS structure for subsequent S_d_^0^ activation and Hg^0^ adsorption. The negative Δ*G* (–68.4 kJ mol^−1^) from ZnS@HgS-S_8_ to ZnS@HgS-S_4_ demostrated its spontaneous convertion process. The *E*_ads_ of Hg^0^ on ZnS@HgS-S_4_ (–73.1 kJ mol^−1^) was higher than those of ZnS@HgS (–26.0 kJ mol^−1^) and ZnS@HgS-S_8_ (–27.9 kJ mol^−1^). These confirm the role of HgS in the activation of S_d_^0^ and further adsorption of Hg^0^, supporting the proposed Hg^0^ self-sustained adsorption mechanism.

## Discussion

**Fig. 6:**
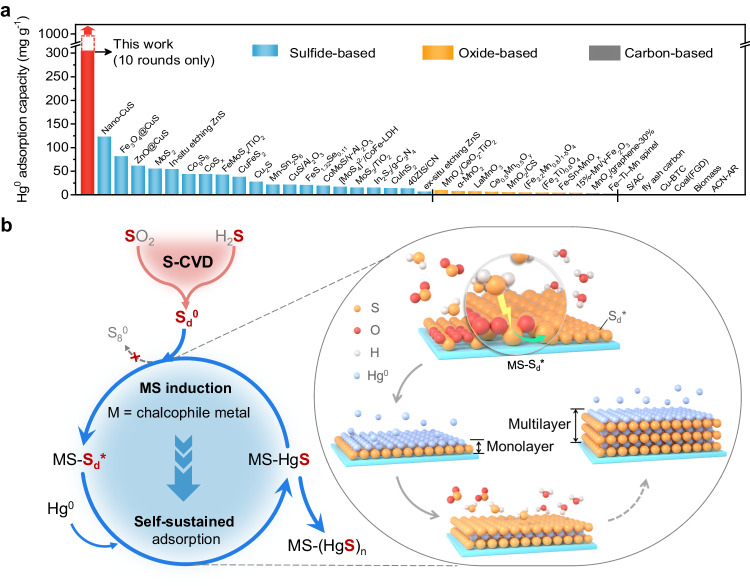
Performance comparison and mechanism schematic. **a** Comparison of Hg^0^ adsorption capacities between Al_2_O_3_@ZnS-S_d_ and other reported adsorbents. The raw data can be found in Supplementary Table 7. **b** Schematic illustration of the proposed in-situ S-CVD technology for self-sustained and multilayer adsorption of Hg^0^. S_d_^0^ is deposited and activated to S_d_^*^ on the Al_2_O_3_@MS by the reaction between flue gas SO_2_ and intermittently added H_2_S; when S_d_^*^ is consumed by Hg^0^, it can be replenished by repeated S-CVD, resulting in self-sustained and multilayer adsorption of Hg^0^.

Figure 6a and Supplementary Table 7 compare the Hg^0^ adsorption capacities of Al_2_O_3_@ZnS-S_d_ with various reported adsorbents. The adsorption capacity of carbon- and oxide-based adsorbents were generally lower than 2 mg g^–1^ and 10 mg g^–1^, respectively, and their performance was severely inhibited by SO_2_. Among the reported sulfide-based adsorbents, nano-CuS exhibited the highest capacity of 122.4 mg g^–1^; however, it decreased to 89.4 mg g^–1^ in the presence of SO_2_ and H_2_O (ref. ^16^). Additionally, our previous work found that under scaled-up conditions, ~1 mm Al_2_O_3_@CuS only had the normalized saturated adsorption capacity of 21.0 mg g^–1^ (ref. ^41^). Another recently reported in-situ acid etching method can boost the adsorption capacity of ZnS to 53.8 mg g^–1^ (ref. ^19^), but it decreased to 1.4 mg g^–1^ under the scale-up conditions (Supplementary Fig. 33). Impressively, the self-sustained Al_2_O_3_@ZnS-S_d_ not only reversed the poisoning effect of SO_2_ but also reached a Hg^0^ adsorption capacity of 303.9 mg g^–1^ (normalized to ZnS coating amount, and with 24-h breakthrough ratio of ~80%) after 10 rounds of reaction, which is over 250, 60, and 8 times higher than the average of reported carbon-, oxide-, and sulfur-based adsorbents, respectively. Moreover, as the self-sustained adsorption process continues, the Hg^0^ adsorption capacity of Al_2_O_3_@ZnS-S_d_ will be increasing, thereby breaking the capacity limitations.

In summary, the Al_2_O_3_@MS-S_d_ adsorbents assisted with intermittent S-CVD can reverse SO_2_ poisoning effects and have great potential for efficient and cost-effective Hg^0^ removal from SO_2_-containing flue gases. This study demonstrates the crucial role of HgS, whether synthesized or formed by Hg^0^ adsorption on Al_2_O_3_@MS-S_d_, in activating of S_d_^0^ into S_d_^*^ (Eqs. 6 and 7). Therefore, this enables the self-sustained adsorption of Hg^0^ on Al_2_O_3_@MS-S_d_ surface-like chain reactions (Eqs. 8–10), realizing the multilayer adsorption (Fig. 6b).

6 $${{{{{{\rm{Al}}}}}}}_{2}{{{{{{\rm{O}}}}}}}_{3}{{{{{\rm{@HgS}}}}}}+{{{{{\rm{S}}}_{{{\rm{d}}}}^0}}}\to {{{{{{\rm{Al}}}}}}}_{2}{{{{{{\rm{O}}}}}}}_{3}{{{{{\rm{@HgS}}}}}}{\mbox{-}}{{{{{{\rm{S}}}}}}}_{{{{{{\rm{d}}}}}}}^{\ast }$$

7 $${{{{{{\rm{Al}}}}}}}_{2}{{{{{{\rm{O}}}}}}}_{3}{{{{{\rm{@MS}}}}}}{\mbox{-}}{{{{{\rm{HgS}}}}}}+{{{{{{\rm{S}}}}}}}_{{{{{{\rm{d}}}}}}}^{0}\to {{{{{{\rm{Al}}}}}}}_{2}{{{{{{\rm{O}}}}}}}_{3}({{{{{\rm{@MS}}}}}}{\mbox{-}}{{{{{\rm{HgS}}}}}}){\mbox{-}}{{{{{{\rm{S}}}}}}}_{{{{{{\rm{d}}}}}}}^{\ast }$$

8 $${{{{{{\rm{Al}}}}}}}_{2}{{{{{{\rm{O}}}}}}}_{3}({{{{{\rm{@MS}}}}}}{\mbox{-}}{{{{{\rm{HgS}}}}}}){\mbox{-}}{{{{{{\rm{S}}}}}}}_{{{{{{\rm{d}}}}}}}^{\ast }+{{{{{{\rm{Hg}}}}}}}^{0}\to {{{{{{\rm{Al}}}}}}}_{2}{{{{{{\rm{O}}}}}}}_{3}{{{{{\rm{@MS}}}}}}{\mbox{-}}{({{{{{\rm{HgS}}}}}})}_{2}$$

9 $${{{{{{\rm{Al}}}}}}}_{2}{{{{{{\rm{O}}}}}}}_{3}{{{{{\rm{@MS}}}}}}{\mbox{-}}{({{{{{\rm{HgS}}}}}})}_{2}+{{{{{{\rm{S}}}}}}}_{{{{{{\rm{d}}}}}}}^{0}\to {{{{{{\rm{Al}}}}}}}_{2}{{{{{{\rm{O}}}}}}}_{3}{{{{{\rm{@MS}}}}}}{\mbox{-}}{({{{{{\rm{HgS}}}}}})}_{2}{\mbox{-}}{{{{{{\rm{S}}}}}}}_{{{{{{\rm{d}}}}}}}^{\ast }$$

10 $${{{{{{\rm{Al}}}}}}}_{2}{{{{{{\rm{O}}}}}}}_{3}{{{{{\rm{@MS}}}}}}{\mbox{-}}{({{{{{\rm{HgS}}}}}})}_{{{{{{\rm{n}}}}}}{-}1}{\mbox{-}}{{{{{{\rm{S}}}}}}}_{{{{{{\rm{d}}}}}}}^{\ast }+{{{{{{\rm{Hg}}}}}}}^{0}\to {{{{{{\rm{Al}}}}}}}_{2}{{{{{{\rm{O}}}}}}}_{3}{{{{{\rm{@MS}}}}}}{\mbox{-}}{({{{{{\rm{HgS}}}}}})}_{{{{{{\rm{n}}}}}}}$$

## Methods

### Materials

The raw materials used in this work were purchased in chemical purity (>99.5%) from Sinopharm Chemical Reagent Co., Ltd and used without purification. Natural sulfide ores (chalcopyrite and sphalerite) were provided by provided by a non-ferrous smelter in Henan, China.

### Preparation of adsorbents

Different metal sulfides (MS, M = Cu, Zn, Cd, In, Hg, Pb, Sn, Mn, Fe, Co, and Ni) were synthesized via a one-step hydrothermal method as the interface for S-CVD and Hg^0^ adsorption. According to our previous study, ~1 mm commercial γ-Al_2_O_3_ pellets were chosen as the support for MS (named Al_2_O_3_@MS) to improve gas mass transfer^41^. Taking Al_2_O_3_@ZnS as an example, firstly, 3.50 mL of H_2_O containing 2.50 mmol of ZnSO_4_·7H_2_O was added into 4.75 g of γ-Al_2_O_3_ pellets followed by 30 min ultrasonic treatment. Then, the mixture was dried at 60 °C for 9 h to obtain Al_2_O_3_@ZnSO_4_. After that, the pellets were poured into 50.0 mL of H_2_O containing 2.50 mmol of Na_2_S·9H_2_O and transferred to a 100 mL Teflon-lined autoclave. After reacting at 120 °C for 12 h, the resultant Al_2_O_3_@ZnS was collected by vacuum filtration, washed with deionized water and ethanol, and dried at 60 °C in an oven for 12 h. Other Al_2_O_3_@MS adsorbents were synthesized in the same way, except that different metal precursors, including CuSO_4_·5H_2_O (2.50 mmol), CdCl_2_ (2.50 mmol), InCl_3_·4H_2_O (1.67 mmol), HgCl_2_ (2.50 mmol), (CH_3_COO)_2_Pb (2.50 mmol), SnCl_2_ (2.50 mmol), MnSO_4_·H_2_O (2.50 mmol), FeCl_3_ (1.67 mmol), CoCl_2_·6H_2_O (2.50 mmol), and NiCl_2_·6H_2_O (2.50 mmol), were used instead of ZnSO_4_·7H_2_O.

### In-situ low-temperature sulfur chemical vapor deposition

The in-situ S-CVD was conducted in a self-made fixed-bed reactor system as shown in Supplementary Fig. 1. To prevent the pre-reaction between H_2_S and SO_2_ in the pipeline (heat protection at 120 °C), a three-way quartz reaction tube was used to introduce different gas components, of which the main tube (10.0 mm inner diameter) was used to feed 5000 ppm SO_2_, and the side tube (4.0 mm inner diameter) embedded in the main reaction tube was used to feed 100 ppm H_2_S. The H_2_S concentration was detected by a gas chromatograph (Agilent GC 8860) equipped with a flame photometric detector. To verify the feasibility of the S-CVD strategy, we used SiO_2_ wafer as substrate to conduct the reaction. The SEM images of SiO_2_-S_d_ exhibited a homogeneous surface with a few grooves and the ESD mapping images showed the uniform distribution of S element (Supplementary Fig. 34a–f). Moreover, the SEM image of the cross-section of SiO_2_-S_d_ showed the formation of a deposition layer (Supplementary Fig. 34g, h).

Specifically, in this work, single S-CVD (15 min) was used to evaluate the enhanced effect of S-CVD on the activities of different Al_2_O_3_@MS adsorbents, while Al_2_O_3_@MS or natural sulfide ores treated with intermittent S-CVD (30 min each round) were applied to assess their self-sustained adsorption performance for Hg^0^.

### Characterizations

The chemical composition of the as-prepared adsorbents was analyzed by X-ray fluorescence spectroscopy on an Epsilon 3X instrument (Netherlands). The thermal stability of the adsorbents was analyzed by a thermogravimetric analyzer (NETZSCH, STA 2500 Regulus) at a heating rate of 5 °C min^−1^ from 20 °C to 500 °C in N_2_ atmosphere. The BET-specific surface area and pore structure were detected by an automatic porosity analyzer apparatus (Quantachrome, Autosorb-iQ, USA). The XRD patterns were obtained on Shimadzu XRD-6100 (Japan) with Cu Kα radiation (scan speed: 8° min^−1^, and 2*θ* range of 10−80°). The SEM images of adsorbents were performed on ZEISS Sigma 300 (Germany), and EDS/mapping images using Oxford Xplore 30 (UK). XPS spectra were determined on an X-ray photoelectron spectrometer (Thermo Scientific ESCALAB Xi+, USA) equipped with a mono Al Kα X-ray source. TEM images were carried out on FEI Tecnai F20. The XPS depth profiling is measured by Ar^+^ ion etching material surface layers at different depths. XAFS spectra of S L-edge and Zn K-edge were detected by synchrotron radiation light sources (see detail in Supplementary Methods 1). Raman spectra were measured on a Raman spectrometer (Horiba LabRAM HR Evolution, Japan) with 532 nm line of Ar^+^ laser for excitation. FTIR spectra were recorded on a Fourier-Transform infrared spectrometer (Nicolet 6700, USA) using the potassium bromide pellet technique. ^13^C NMR spectra were performed on a nuclear magnetic resonance spectrometer (Bruker Avance Neo 400WB, Germany).

### Gaseous mercury adsorption assessment

Hg^0^ adsorption performances of as-prepared adsorbents were assessed in the same reactor system. The Hg^0^ vapor was obtained by an Hg^0^ permeation device (VICI Metronics) loaded in a U-shaped glass tube. The H_2_O vapor was produced by a steam generator. The concentrations of both Hg^0^ and H_2_O were controlled by adjusting the water bath temperature and carrier gas (N_2_) flow rate. Other gas component including O_2_, SO_2_, and NO, were obtained directly from compressed gas in cylinders. The gas components of the simulated flue gas, including Hg^0^ (1.5−2.5 mg m^−3^), SO_2_ (5000−60,000 ppm, when used), and H_2_O (4%, when used), O_2_ (5%, when used), and NO (100 ppm, when used) were mixed evenly before entering the fixed-bed reactor. The total flow rate was controlled at 300−360 mL min^−1^, the adsorbent mass used in each experiment was 0.3−1.0 g. The detailed experiment conditions were summarized in Supplementary Table 8. Lumex RA 915+ was used to record the inlet and outlet Hg^0^ concentrations and before each experiment, the inlet concentration should remain stable (±0.05 μg m^−3^) for more than 10 min. The exhaust gas was absorbed by 0.1 mol L^−1^ potassium permanganate solution and activated carbon before discharged.

The Hg^0^ removal efficiency (*η*, %) and Hg^0^ adsorption capacity (*Q*, mg·g^−1^) were calculated according to following equations:

11 $${{{{{\rm{\eta }}}}}}=\frac{{{{\mbox{Hg}}}}_{{{\mbox{in}}}}^{0}{{{-}}}{{{\mbox{Hg}}}}_{{{\mbox{out}}}}^{0}}{{{{\mbox{Hg}}}}_{{{\mbox{in}}}}^{0}}\times 100\%$$

12 $${{\mbox{Q}}}={\int }_{{{{\mbox{t}}}}_{1}}^{{{{\mbox{t}}}}_{2}}\frac{{{{\mbox{Hg}}}}_{{{\mbox{in}}}}^{0}{{{{-}}}{{\mbox{Hg}}}}_{{{\mbox{out}}}}^{0}}{{{\mbox{m}}}}\times {{\mbox{fdt}}}$$

where Hg^0^_in_ and Hg^0^_out_ (mg m^−3^) are the inlet and outlet Hg^0^ concentrations, respectively, *m* (g) is the adsorbent mass, *f* (mL min^−1^) represents the total flow rate, and *t* (min) donates the adsorption time.

Hg^0^-TPD experiments were conducted to identify the Hg^0^ species adsorbed on the adsorbent surface and its stability. A certain amount of spent adsorbent was heated from 50 to 450 °C with a heating rate of 5 °C min^−1^ in pure N_2_ to desorb the adsorbed mercury. The signal of desorbed Hg^0^ was also detected by Lumex RA 915+.

### Kinetics and theory calculation

Hg^0^ adsorption kinetic models and theoretical calculations of S_d_^0^ activation and Hg^0^ adsorption behavior are detailed in Supplementary Methods 2 and 3.

### Supplementary information

Supplementary Information

Peer Review File

## Data Availability

All data supporting the findings of this study are available within the paper and the Supplementary information files or available from the corresponding author upon request.
