# Supplementary material for: In-situ low-temperature sulfur CVD on metal sulfides with SO2 to realize self-sustained adsorption of mercury
Source: Nat Commun. 2024 Apr 18;15:3362. doi: 10.1038/s41467-024-47725-3 (PMC11026451; doi:10.1038/s41467-024-47725-3)
Supplement: Supplementary file 1 — Supplementary Information [file 41467_2024_47725_MOESM1_ESM.pdf]

Supplementary Information

In-situ Low-temperature Sulfur CVD on Metal Sulfides with SO_2 to Realize Self-sustained Adsorption of Mercury

Qinyuan Hong[1], Haomiao Xu[1]*, Xiaoming Sun[1], Jiaxing Li[1], Wenjun Huang[1], Zan Qu[1,2]*, Lizhi Zhang[1,3], Naiqiang Yan[1,2]*

[1] School of Environmental Science and Engineering, Shanghai Jiao Tong University, Shanghai 200240, China. [2] Shanghai Institute of Pollution Control and Ecological Security, Shanghai 200092, China. [3] Key Laboratory of Pesticide & Chemical Biology of Ministry of Education Institute of Applied & Environmental Chemistry College of Chemistry, Central China Normal University, Wuhan 430079, China. These authors contributed equally: Qinyuan Hong, Haomiao Xu. **Email:* xuhaomiao@sjtu.edu.cn; quzan@sjtu.edu.cn; nqyan@sjtu.edu.cn

Supplementary Methods 1

Characterization of X-ray absorption fine spectra (XAFS). The XAFS of S L-edge were determined by beamlines MCD-A and MCD-B (Soochow Beamline for Energy Materials) at the National Synchrotron Radiation Laboratory (NSRL), and the Zn K-edge spectra were measured in transmission mode at the beamline 1W2B of Beijing Synchrotron Radiation Facility (BSRF). EXAFS fitting data were accomplished using the Athena and Artemis modules in the Demeter data analysis package [1]. The k^3-weighted χ(k) data were Fourier transformed after applying a HanFeng window function (Δk = 1.0). The EXAFS of the Zn foil was fitted and the obtained amplitude reduction factor (S_0^2 = 0.865) was used in the EXAFS analysis of the sample to obtain the coordination numbers (CNs) of the Zn–S scattering path.

Supplementary Methods 2

Kinetic adsorption model. Different kinetic models, including pseudo-first-order model[2], pseudo-second order model[3], intra-particle diffusion model[4], and Elovich model[5], were applied to analyze the Hg^0 adsorption rate of as-prepared adsorbents. The model with the highest coefficient factor (R^2) was considered as the optimal model to describe the kinetic behavior of Hg^0 adsorption on the adsorbents. The related kinetic models are as follows:

Pseudo-first-order: $\ln(q_e - q_t) = \ln q_e - k_1 t$ (S1)

Pseudo-second order: $t/q_t = 1/(k_2 q_e^2) + t/q_e$ (S2)

Intra-particle diffusion: $q_t = k_p t^{1/2} + C$ (S3)

Elovich: $q_t = 1/\beta \cdot \ln(\alpha\beta) + \ln t/\beta$ (S4)

where q_t and q_e (mg g^{-1}) are the mercury adsorption capacities at time t and equilibrium time, respectively, and t (min) is the reaction time. k_1 (min^{-1}), k_2 (g mg^{-1} min^{-1}) and k_p (mg g^{-1} $min^{-1/2}$) donate the rate constants of pseudo-first-order, pseudo-second-order and particle-internal-diffusion model, respectively. α (mg g^{-1} min^{-1}) represents the initial rate and β (min^{-1}) is related to the extent of surface coverage and the activation energy for chemisorption.

Supplementary Methods 3

DFT calculations. The theoretical calculations were performed by Vienna Ab initio Simulation Package (VASP 6.1.0) on the basis of DFT. The exchange-correlation potential is described by the generalized gradient approximation of Perdew-Burke-Ernzerhof (GGA-PBE)[6]. The projector augmented-wave (PAW) method is employed to treat interactions between ion cores and valence electrons. The plane-wave cutoff energy was fixed to 450 eV. Given structural models were relaxed until the Hellmann–Feynman forces smaller than 0.02 eV $Å^{-1}$ and the change in energy smaller than 10^{-5} eV was attained. Grimme's DFT-D3 methodology was used to describe the dispersion interactions among all the atoms in adsorption models.

The Gibbs free energy change is defined as:

$$\Delta G = \Delta E + \Delta ZPE - T\Delta S \quad (S5)$$

where ΔE is the electronic energy calculated with VASP, ΔZPE and ΔS are the zero-point energy difference and the entropy change between the products and reactants, respectively, and T is the temperature (298.15 K).

The adsorption energy (E_{ads}) was calculated by the Born–Oppenheimer energy difference according to following equation:

$$E_{ads} = E_{total} - E_{substrate} - E_{adsorbate} \quad (S6)$$

where E_{total}, $E_{substrate}$ and $E_{adsorbate}$ represent the energies of the substrate with adsorbed molecule, raw substrate, and adsorbate, respectively.

Supplementary Methods 4

Preparation of in-situ etching ZnS and Al_2O_3@ZnS. The in-situ etching ZnS was synthesized according to the published paper[7]. Typically, 0.01 mol of $ZnSO_4\cdot7H_2O$ was dissolved in 50 mL of acid solution (0.368 mol L^{-1} of H_2SO_4), and 0.01 mol of $Na_2S\cdot9H_2O$ was dissolved in 50 mL of deionized water separately. Then, the Na_2S solution was added into the $ZnSO_4$ solution to obtain a light-yellow precipitate. The precipitate was separated by centrifugation and washed several times with deionized water and ethanol. Finally, the precipitate was dried in vacuum at 60 °C for 12 h to obtain in-situ etching ZnS.

The in-situ etching Al_2O_3@ZnS was synthesized by a similar method. The difference is that 50 mL of $ZnSO_4$ solution was first mixed with 20 g of 1−2 mm Al_2O_3 pellets and ultrasonicated for 30 min and then dried to obtain Al_2O_3@$ZnSO_4$.

Supplementary Figures

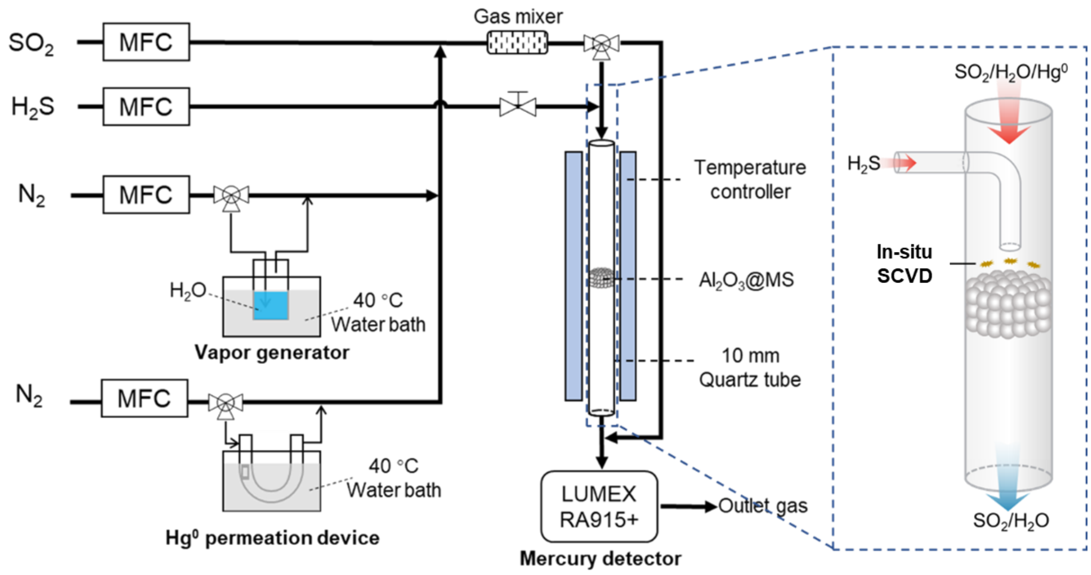

Supplementary Fig. 1 Schematic diagram of the self-made fixed-bed adsorption and the in-situ S-CVD system**.**

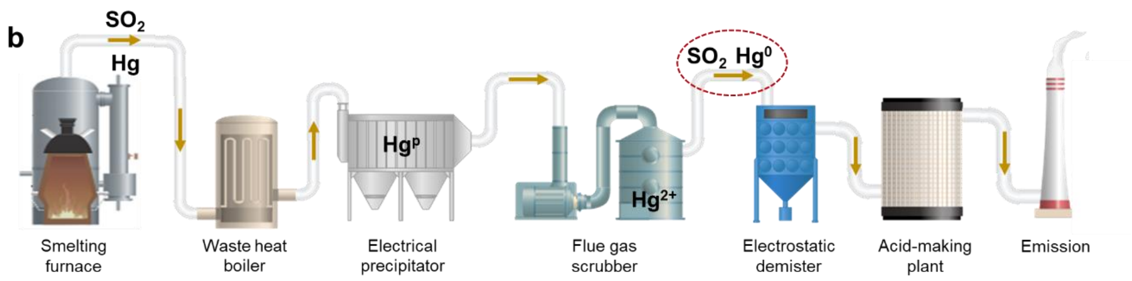

Supplementary Fig. 2 Application of the S-CVD method to practical non-ferrous smelting processes.

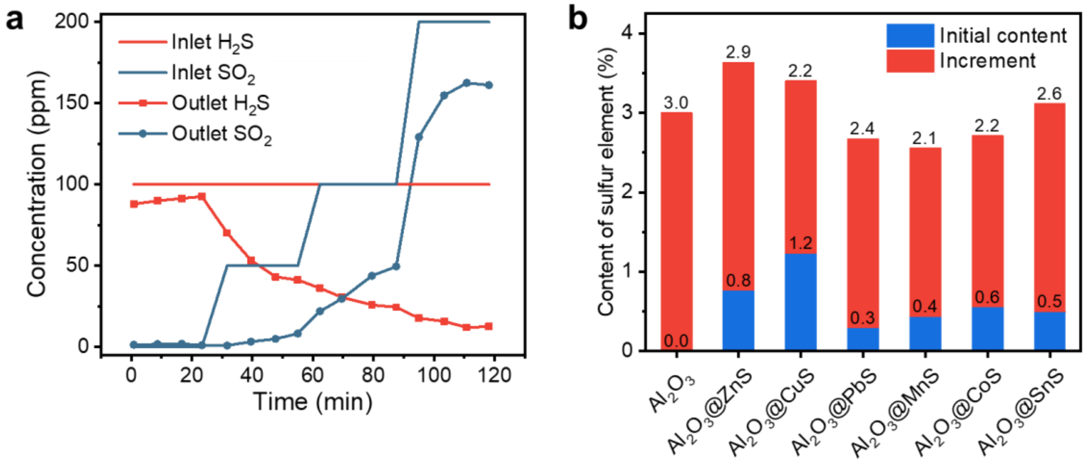

Supplementary Fig. 3 Variation of different sulfur species during S-CVD process. a Variation of inlet and outlet concentrations of H_2S and SO_2 during the S-CVD process on γ-Al_2O_3. **b** Changes of sulfur content in different adsorbents after 180 min S-CVD.

Supplementary Discussion

Variation of different sulfur species during S-CVD process. During the S-CVD process, we monitored concentrations of H_2S and SO_2 at the inlet and outlet. Initially, the outlet H_2S concentration maintained at around 92 ppm (inlet 100 ppm) before the introduction of SO_2. However, as the inlet SO_2 concentration increased to 200 ppm, the outlet H_2S concentration gradually decreased to 12.2±0.2 ppm, and the outlet SO_2 concentration was detected as 157.9±0.7 ppm.

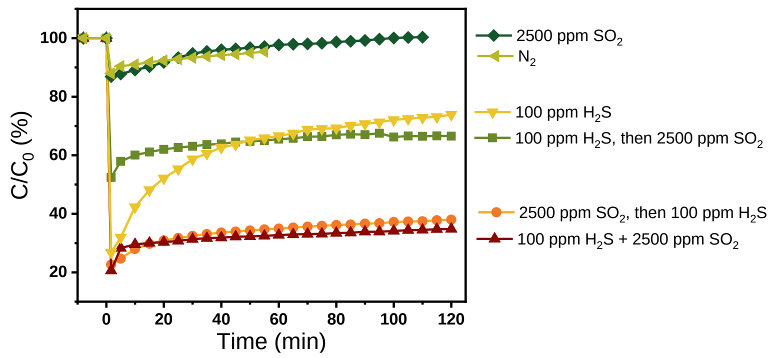

Supplementary Fig. 4 The effect of the addition sequence of H_2S and SO_2 on the Hg^0 adsorption performance of Al_2O_3@ZnS-S_d during S-CVD process. Reaction conditions: sorbent mass = 0.3 g, total flow rate = 360 mL min^{-1}, S-CVD time = 15 min, Hg^0 concentration = (1.5±0.05) mg m^{-3}, reaction temperature = 80 °C.

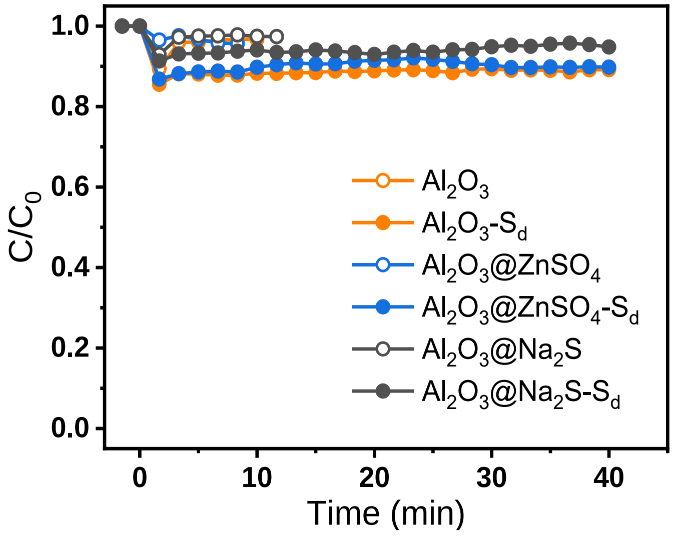

Supplementary Fig. 5 Hg^0 adsorption performance of raw and activated Al_2O_3, Al_2O_3@$ZnSO_4$, and Al_2O_3@Na_2S. Reaction conditions: sorbent mass = 0.3 g, SO_2 concentration = 5000 ppm (during S-CVD process), H_2S concentration = 100 ppm (during S-CVD process), S-CVD time = 15 min, Hg^0 concentration = (1.5±0.05) mg m^{-3}, reaction temperature = 80 °C, and total flow rate = 360 mL min^{-1}.

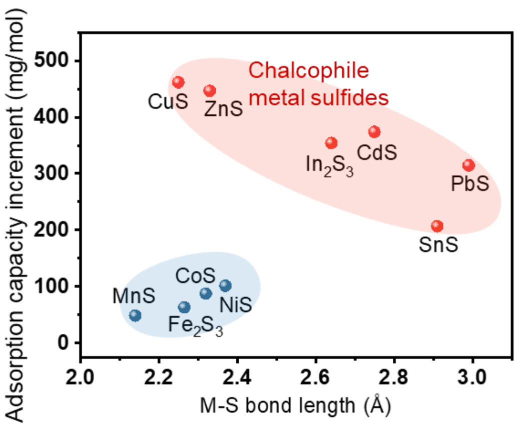

Supplementary Fig. 6 The tendency between the adsorption capacity increment and the metal-sulfur bond length of different metal sulfides.

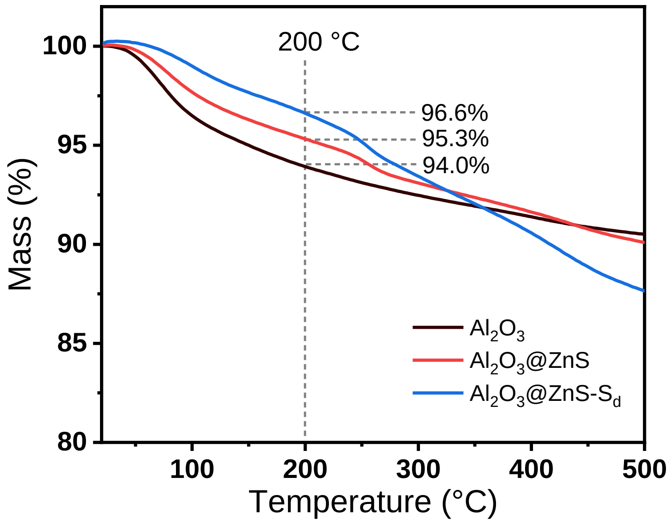

Supplementary Fig. 7 Thermogravimetric analysis of Al_2O_3, Al_2O_3@ZnS, and Al_2O_3@ZnS-S_d.

Supplementary Discussion

Thermogravimetric analysis (TGA). The results showed that the mass loss of Al_2O_3@ZnS-S_d at 200 °C was reduced by 2.6% and 1.3% compared with that of Al_2O_3 and Al_2O_3@ZnS, respectively (Supplementary Fig. 7), reflecting the high thermal stability of ZnS and S_d^0 deposited on Al_2O_3@ZnS.

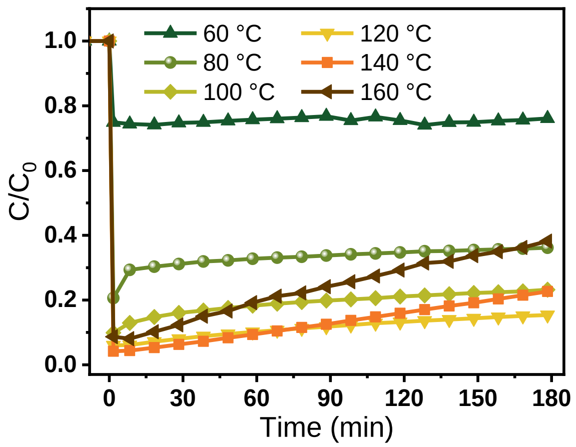

Supplementary Fig. 8 Hg^0 adsorption curves of Al_2O_3@ZnS-S_d at different temperatures. Reaction conditions: sorbent mass = 0.3 g, SO_2 concentration = 5000 ppm (during S-CVD process), H_2S concentration = 100 ppm (during S-CVD process), S-CVD time = 15 min, Hg^0 concentration = (1.5±0.05) mg m^{-3}, and total flow rate = 360 mL min^{-1}.

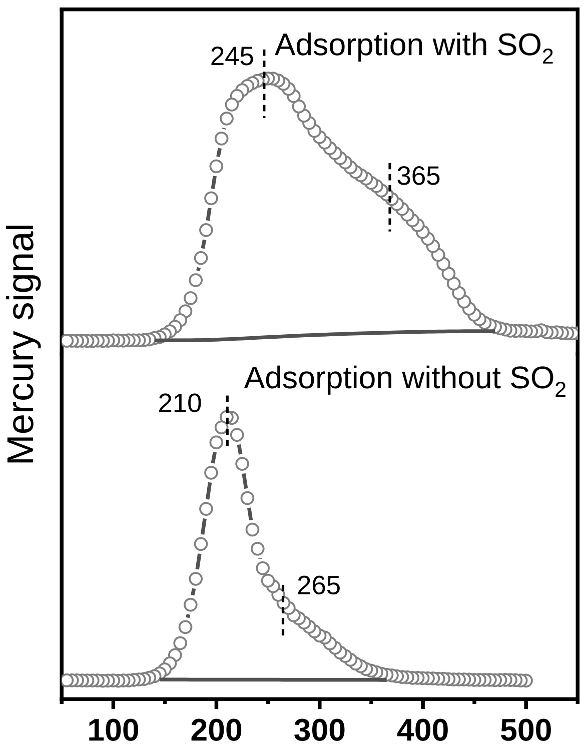

Supplementary Fig. 9 Hg^0 Temperature programmed desorption (Hg^0-TPD) curves of spent Al_2O_3@ZnS-S_d after Hg^0 adsorption with or without the presence of SO_2.

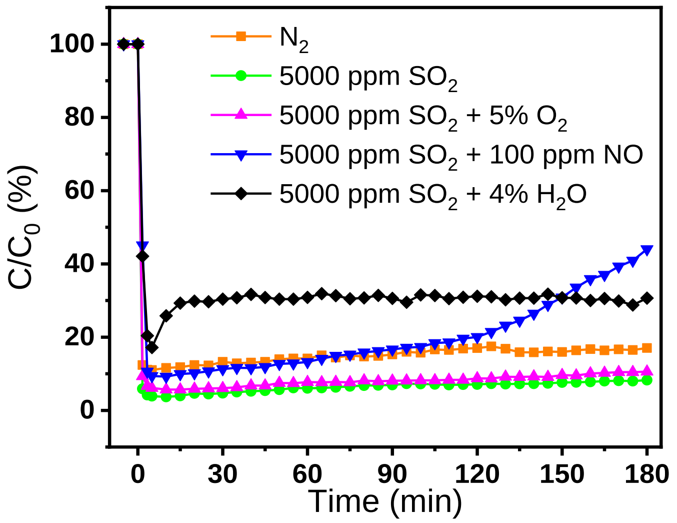

Supplementary Fig. 10 Effect of different gas components on Hg^0 adsorption performance over Al_2O_3@ZnS-S_d. Reaction conditions: sorbent mass = 0.3 g, SO_2 concentration = 5000 ppm (during S-CVD process), H_2S concentration = 100 ppm (during S-CVD process), S-CVD time = 15 min, Hg^0 concentration = (1.5±0.05) mg m^{-3}, reaction temperature = 120 °C, and total flow rate = 360 mL min^{-1}.

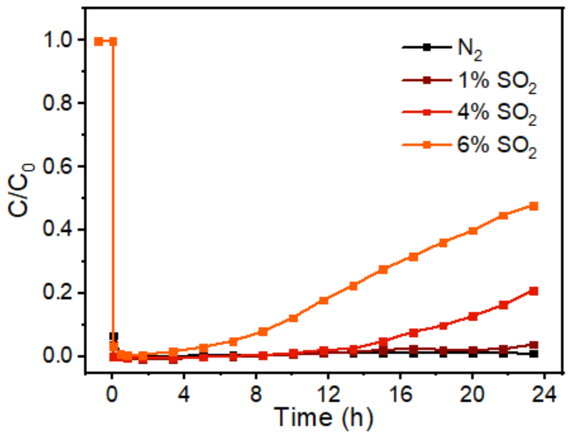

Supplementary Fig. 11 The effect of SO_2 concentration on the Hg^0 adsorption performance of Al_2O_3@CuS without S-CVD. Reaction conditions: sorbent mass = 5 g, temperature = 80 °C, flow rate = 200 mL min^{-1}, and $[Hg^0]_{in}$ = 2400−2500 μg m^{-3}.

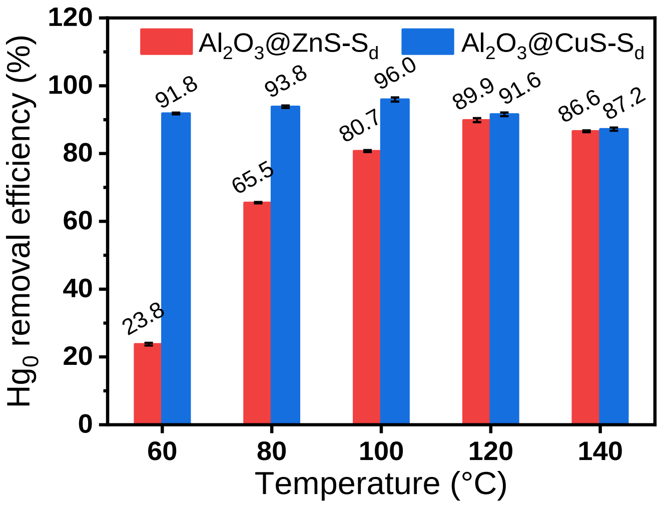

Supplementary Fig. 12 Temperature effect on the Hg^0 removal efficiency of Al_2O_3@ZnS-S_d and Al_2O_3@CuS-S_d. The error bars represent the standard deviation of three parallel tests. Reaction conditions: sorbent mass = 0.3 g, SO_2 concentration = 5000 ppm (during S-CVD process), H_2S concentration = 100 ppm (during S-CVD process), S-CVD time = 15 min, Hg^0 concentration = (1.5±0.05) mg m^{-3}, and total flow rate = 360 mL min^{-1}.

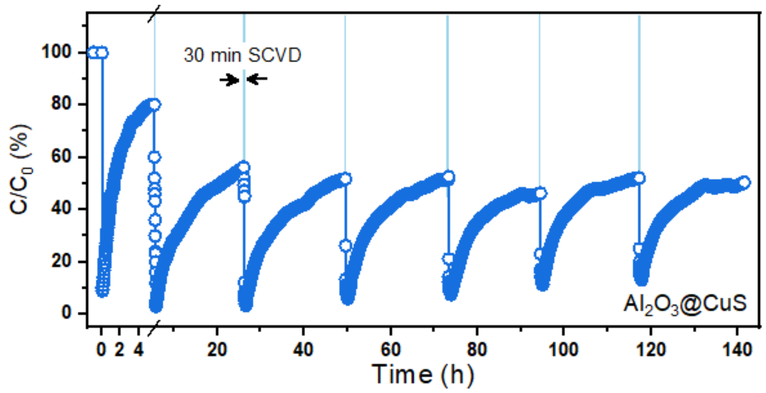

Supplementary Fig. 13 Self-sustained Hg^0 adsorption curve of Al_2O_3@CuS-S_d. Reaction conditions: sorbent mass = 0.4 g, temperature = 60 °C, [Hg^0] = (2.5±0.05) mg m^{-3}, [SO_2] = 5000 ppm, [H_2O] = 4%, [H_2S] = 100 ppm (30 min 24 h^{-1}), and total flow rate = 300 mL min^{-1}.

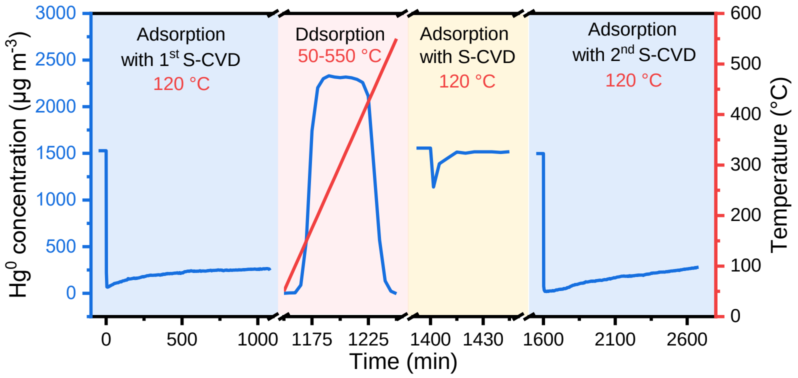

Supplementary Fig. 14 Effect of the Hg^0 adsorption-desorption cycle on the performance of Al_2O_3@ZnS-S_d. Reaction conditions: adsorbent weight = 0.4 g, adsorption temperature = 120 °C, desorption temperature = 50−550 °C (heating rate = 5 °C min^{-1}), S-CVD time = 30 min (5000 ppm SO_2 + 100 ppm H_2S), total flow rate = 360 mL min^{-1}.

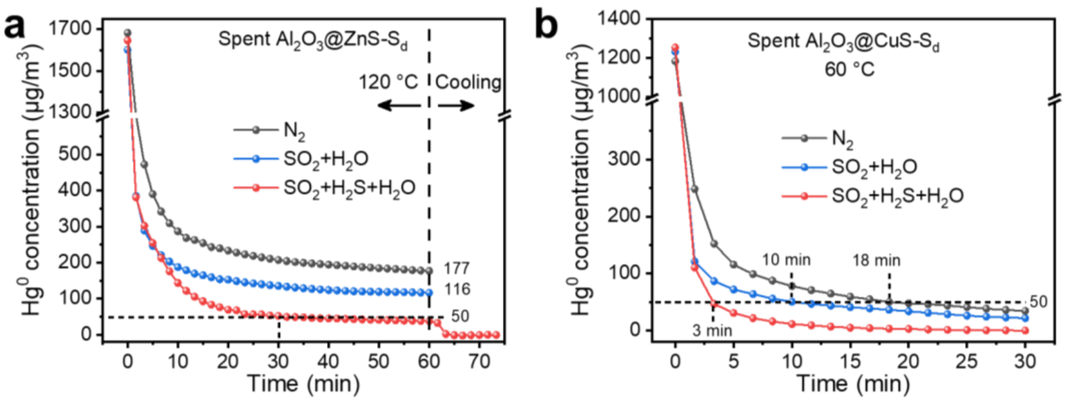

Supplementary Fig. 15 Hg^0 re-emission experiments. a Spent Al_2O_3@ZnS-S_d and **b** spent Al_2O_3@CuS-S_d. Reaction conditions: sorbent mass = 0.4 g, Hg^0 concentration = 0 mg m^{-3}, SO_2 concentration = 5000 ppm (when used), H_2O concentration = 4% (when used), H_2S concentration = 100 ppm (when used), and total flow rate = 300 mL min^{-1}.

Supplementary Discussion

Hg^0 re-emission results. The results showed that after cutting off the injection of Hg^0, at the reaction temperature of Al_2O_3@ZnS-S_d (120 °C), the Hg^0 concentration reduced to 177 μg m^{-3} and 116 μg m^{-3} under N_2 and SO_2+H_2O conditions, respectively, within a 60 min purge (Supplementary Fig. 15a). While, with the addition of 100 ppm of H_2S, the Hg^0 concentration can decrease to lower than 0.05 mg m^{-3} (emission standard for non-ferrous smelting flue gas in China) in 30 min, and once the temperature dropped to room temperature, the Hg^0 concentration rapidly decreased to 0 (Supplementary Fig. 15a). For Al_2O_3@CuS-S_d reacted at 60 °C, the Hg^0 concentration reduced to 50 μg m^{-3} within 18, 10, and 3 min for N_2, SO_2+H_2O, and SO_2+H_2S+H_2O purging conditions, respectively (Supplementary Fig. 15b).

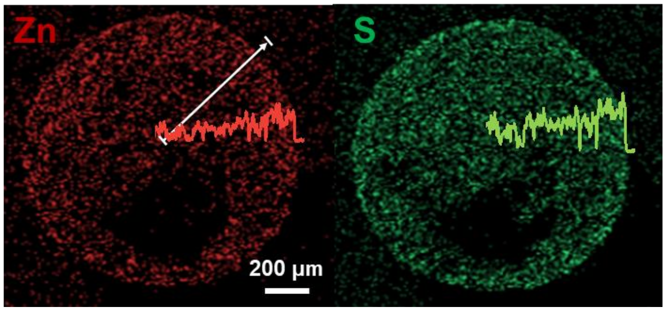

Supplementary Fig. 16 EDS mapping images of the cross section of Al_2O_3@ZnS. Inset: line scanning results of the selected position.

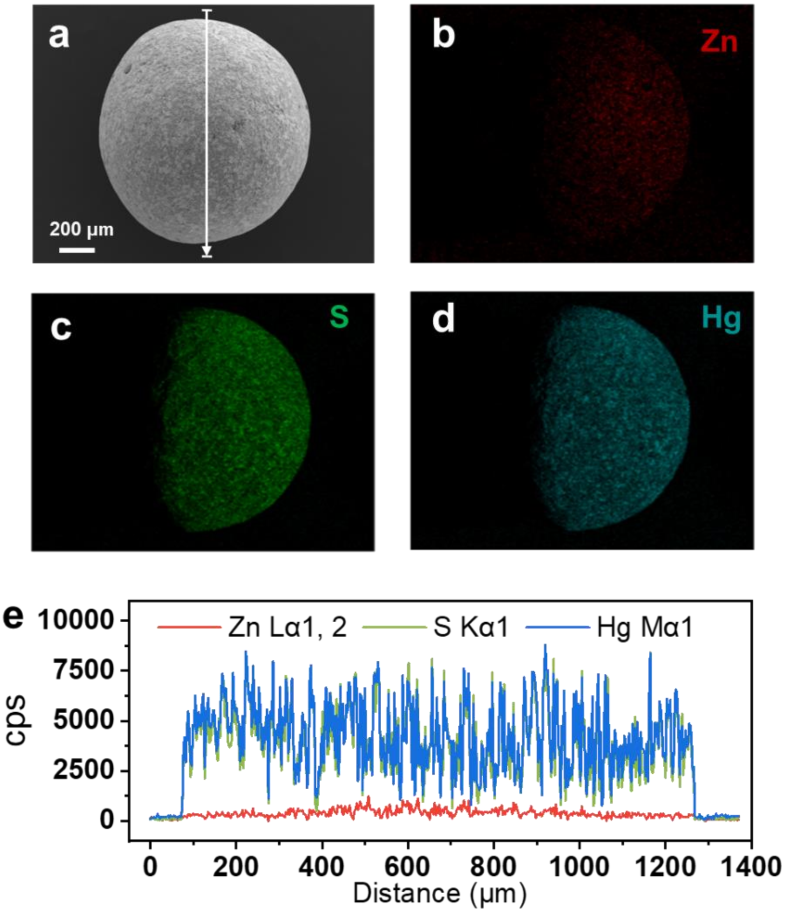

Supplementary Fig. 17 (a) SEM and (b-d) EDS mapping images of the surface of spent Al_2O_3@ZnS-S_d; (e) Line scanning results of the selected position in a.

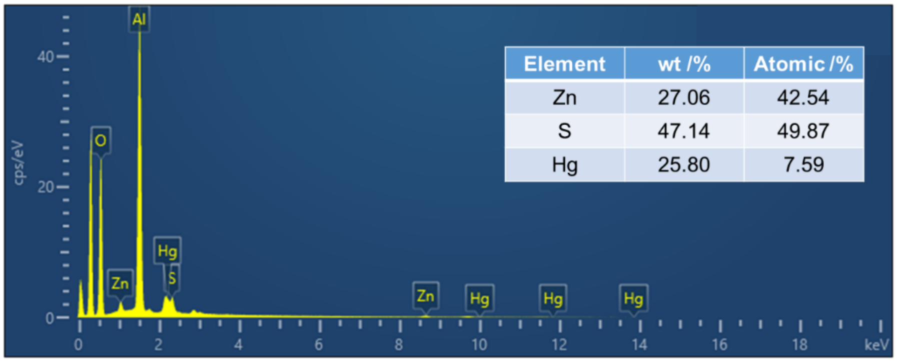

Supplementary Fig. 18 EDS result of the cross section of spent Al_2O_3@ZnS-S_d pellet.

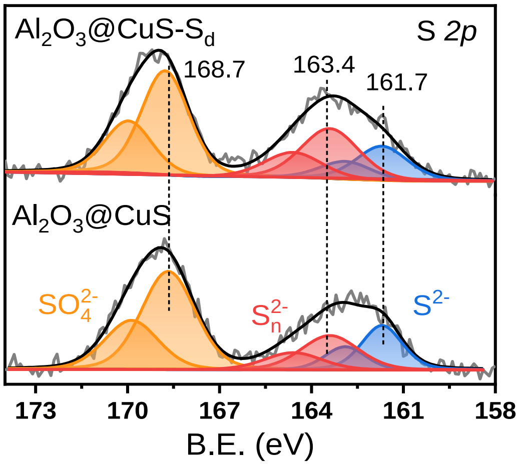

Supplementary Fig. 19 S *2p* XPS spectra of Al_2O_3@CuS and Al_2O_3@CuS-S_d.

Supplementary Discussion

XPS results of Al_2O_3@CuS and Al_2O_3@CuS-S_d. Owing to the variable valence state of Cu in CuS, there occurred the binding energy of S_n^{2-} in Al_2O_3@CuS (bottom layer in Supplementary Fig. 19), which explained its higher Hg^0 adsorption performance than other Al_2O_3@MS adsorbents (Fig. 2c). After S-CVD, the proportion of S_n^{2-} in Al_2O_3@CuS-S_d increased from 21.6% to 29.7% (upper layer in Supplementary Fig. 19, Supplementary Table 5), thus further enhancing its Hg^0 adsorption capacity.

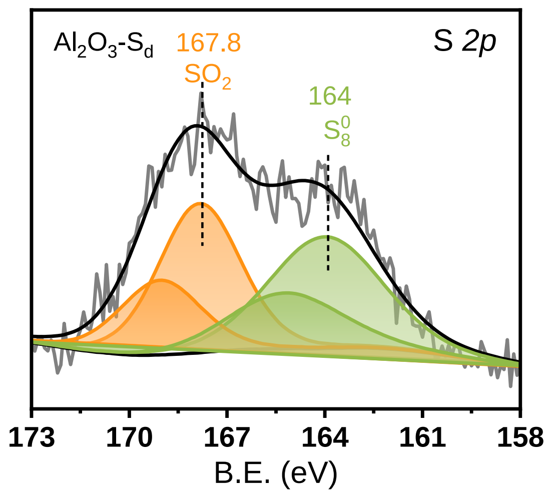

Supplementary Fig. 20 S 2*p* XPS spectrum of Al_2O_3-S_d.

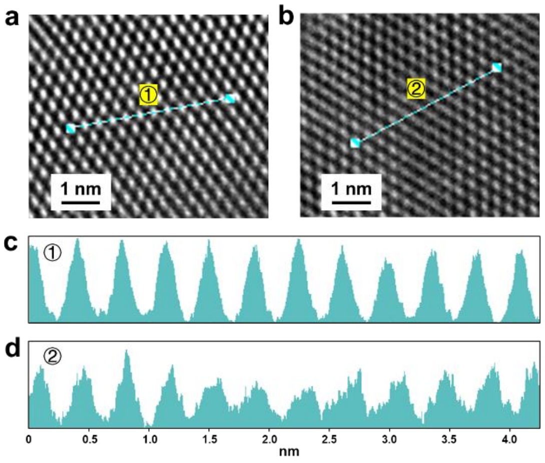

Supplementary Fig. 21 STEM-HAADF images and corresponding simulated elemental map. STEM-HAADF images of **a** ZnS and **b** ZnS-S_d. **c** and **d** The corresponding simulated elemental map of zinc atoms marked in a and b.

Supplementary Discussion

TEM images analysis. The STEM-HAADF (scanning-transmission electron microscopy equipped with a high-angle annular dark field detector) image of ZnS exhibited intact and well-ordered Zn atoms (Supplementary Fig. 21a), and simulated elemental map of atoms showed homogeneous atomic intensity (Supplementary Fig. 21c). However, the STEM-HAADF image of ZnS-S_d showed a decrease in the contrasts of zinc atoms (Supplementary Fig. 21b and d). This indicates the deposition of S_d^0 on ZnS surface resulted in the formation of Zn defects, which is in line with the results of XPS spectra that demonstrated the generation of unsaturated coordinated S_n^{2-} after S_d^0 deposition of Al_2O_3@ZnS.

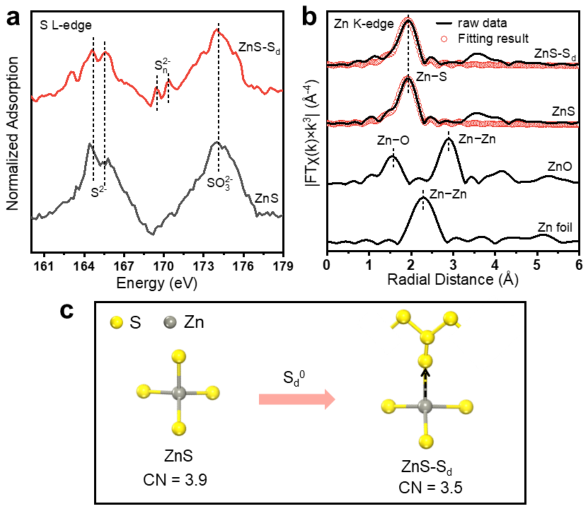

Supplementary Fig. 22 XAFS results of ZnS and ZnS-S$_d$. a S L-edge XANES patterns of ZnS and ZnS-S$_d$. **b** Zn K-edge EXAFS patterns of ZnS, ZnS-S$_d$, Zn foil, and ZnO. **c** Scheme illustration of the coordination number change from ZnS to ZnS-S$_d$.

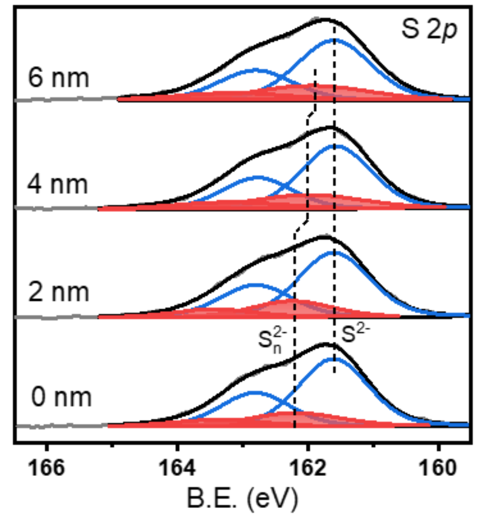

Supplementary Fig. 23 The XPS spectra of ZnS-S_d at different Ar^+ etching depth.

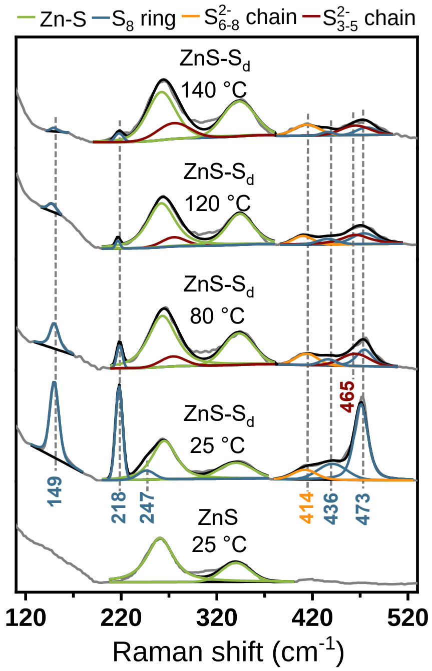

Supplementary Fig. 24 Raman spectra of ZnS and in-situ Raman spectra of ZnS-S at temperature range of 25−140 °C.

Supplementary Discussion

Dynamic variation of sulfur species in ZnS-S_d. The in-situ heating Raman spectra of the resulting ZnS-S_d showed that with temperature increased from 25 to 140 °C, the intensities of the characteristic peaks of S_8 gradually decreased, while the vibration modes of S_n^{2-} chain increased[8]. Yang et al. found that long-chain S_n^{2-} had negligible adsorption ability, while short-chain S_n^{2-} had a high affinity for Hg^0 (ref. [9]). As shown, the ratio of short-chain $S_{3\text{-}5}$ (465 cm^{-1}) to long-chain $S_{6\text{-}8}$ (414 cm^{-1}) increased from 1.4 at 80 °C to 2.2 at 120 °C, and then decreased to 0.9 at 140 °C[10]. This reveals that short-chain $S_{3\text{-}5}^{2-}$ gradually becomes the dominant sulfur species on ZnS surface at elevated temperature from 25 to 120 °C, and explains the decrease in Hg^0 adsorption activity at temperatures above 120 °C.

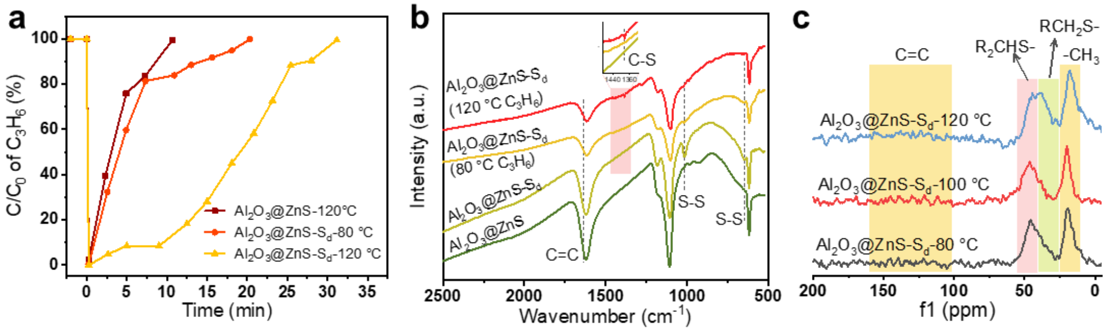

Supplementary Fig. 25 Demonstration of polysulfide chain generation by cross-linking method. a C_3H_6 adsorption breakthrough curves of Al_2O_3@ZnS-S_d at 80 and 120 °C. Reaction conditions: sorbent weight = 0.3 g, flow rate = 500 mL min^{-1}, C_3H_6 concentration = 4000 ppm. **b** Fourier transform infrared spectroscopy (FTIR) spectra of Al_2O_3@ZnS, Al_2O_3@ZnS-S_d and Al_2O_3@ZnS-S_d after adsorption of C_3H_6 at 80 and 120 °C. Inset: localized enlargement of the red region. **c** ^{13}C nuclear magnetic resonance (NMR) spectra of Al_2O_3@ZnS-S_d after adsorption of C_3H_6 at 80, 100 and 120 °C. The highlighted regions represent different carbon containing structure.

Supplementary Discussion

Cross-linking reaction between Al_2O_3@ZnS-S_d and propylene (C_3H_6). As presented in Supplementary Fig. 25a, the cross-linking amount of C_3H_6 over Al_2O_3@ZnS probably due to its porous structure. The adsorption amount over Al_2O_3@ZnS-S_d was calculated as 0.16 and 1.30 mmol at 80 and 120 °C, respectively, when subtracting out the background of Al_2O_3@ZnS. Moreover, as shown in Supplementary Fig. 25b, after Al_2O_3@ZnS-S_d cross-linked with C_3H_6 at 120 °C, the Fourier transform infrared spectroscopy (FTIR) spectra exhibited the characteristic peak of C–S vibration at 1385 cm^{-1} (ref. [11]), while the peak of S–S vibration at 1016 cm^{-1} gradually disappeared[12]. The ^{13}C nuclear magnetic resonance (NMR) results showed the formation of –CH_2S– at 25–38 ppm and –CHS– at 43–58 ppm[13] (Supplementary Fig. 25c); in addition, no peaks related to C=C were found in both FTIR and ^{13}C NMR spectra[14,15]. Thus, this verifies the existence of S_x chain on Al_2O_3@ZnS-S_d under thermal induction.

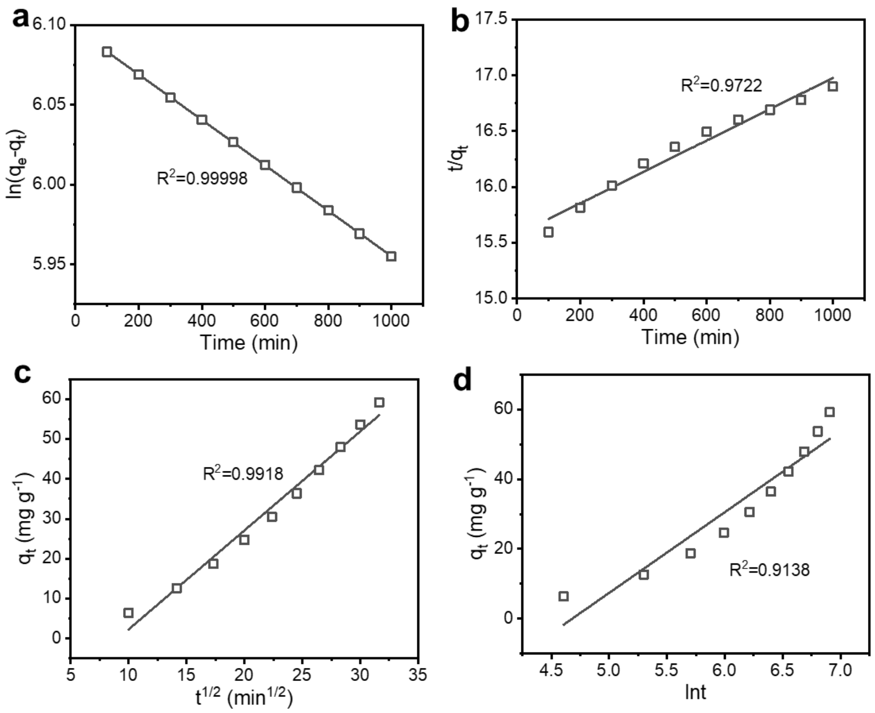

Supplementary Fig. 26 Kinetic analysis of Hg^0 adsorption over Al_2O_3@ZnS-S_d. **a** pseudo-first-order model, **b** pseudo-second-order model, **c** intraparticle diffusion model, and **d** Elovich model.

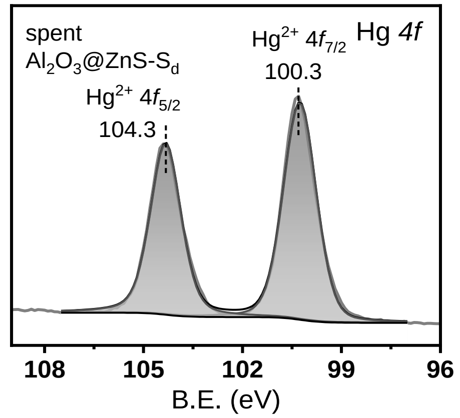

Supplementary Fig. 27 Hg 4*f* XPS spectrum of spent Al_2O_3@ZnS-S_d.

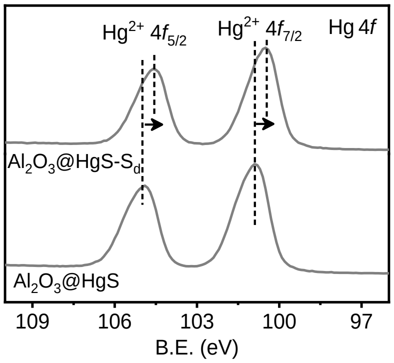

Supplementary Fig. 28 Hg 4*f* XPS spectra of Al_2O_3@HgS and Al_2O_3@HgS-S_d.

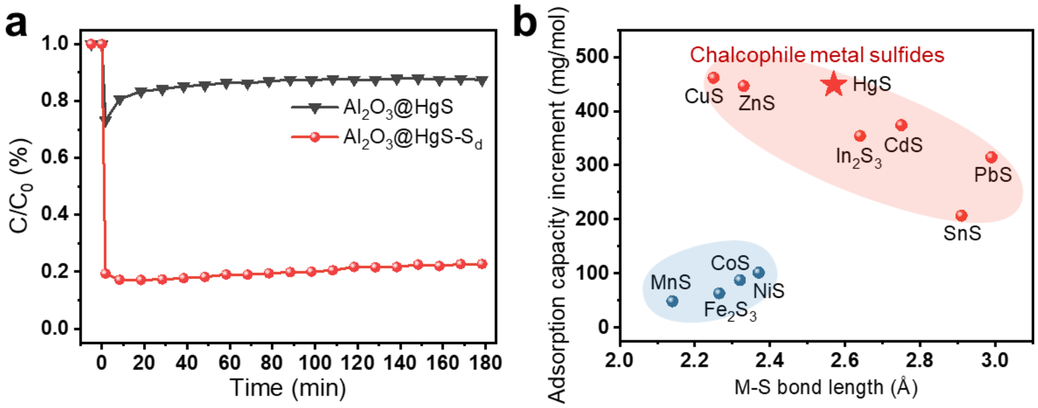

Supplementary Fig. 29 Activation performance of HgS on S_d^0. a Hg^0 adsorption curves of Al_2O_3@HgS and Al_2O_3@HgS-S_d. Reaction conditions: sorbent mass = 0.3 g, SO_2 concentration = 5000 ppm (during S-CVD process), H_2S concentration = 100 ppm (during S-CVD process), S-CVD time = 15 min, Hg^0 concentration = (1.5±0.05) mg m^{-3}, reaction temperature = 80 °C, and total flow rate = 360 mL min^{-1}. **b** The tendency between the adsorption capacity increment and the metal-sulfur bond length of different metal sulfides (including HgS).

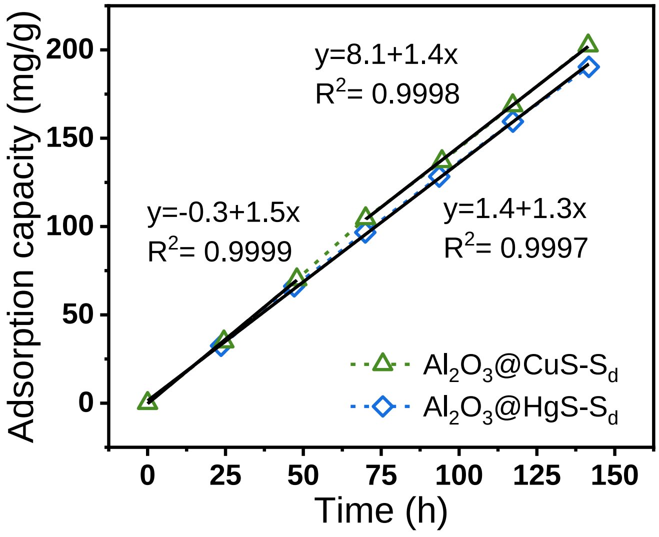

Supplementary Fig. 30 Linear fitting of the adsorption rates of Al_2O_3@CuS-S_d and Al_2O_3@Hg.

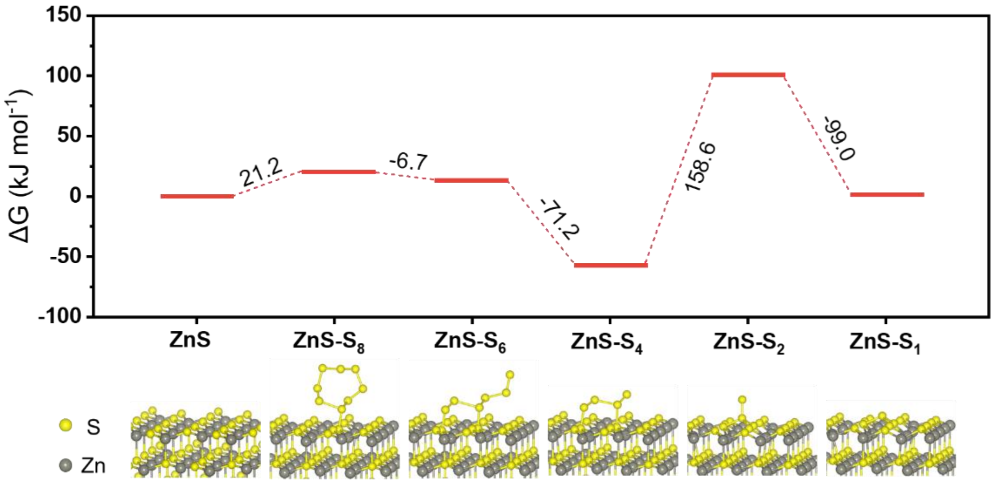

Supplementary Fig. 31 Gibbs free energy of S_8 (S_d^0) activation on ZnS(111) surface.

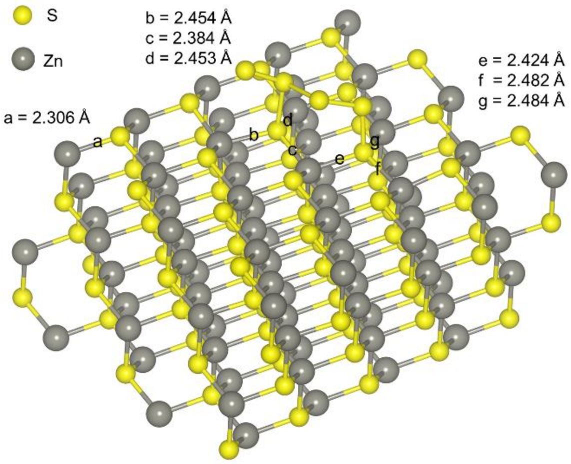

Supplementary Fig. 32 Zn–S bond length in ZnS and ZnS-S_4 surface.

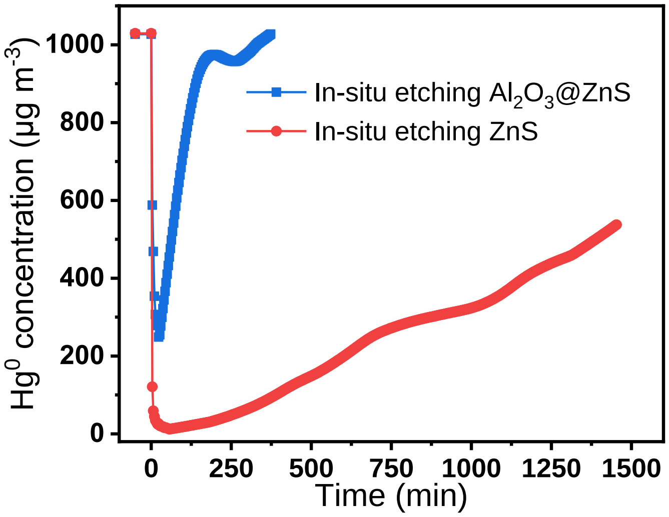

Supplementary Fig. 33 Hg^0 adsorption performance of in-situ etching ZnS and Al_2O_3@ZnS. Reaction conditions: sorbent mass = 20 mg of in-situ etching ZnS or 0.3 g of in-situ etching Al_2O_3@ZnS, reaction temperature = 120 °C, and total flow rate = 360 mL min^{-1}.

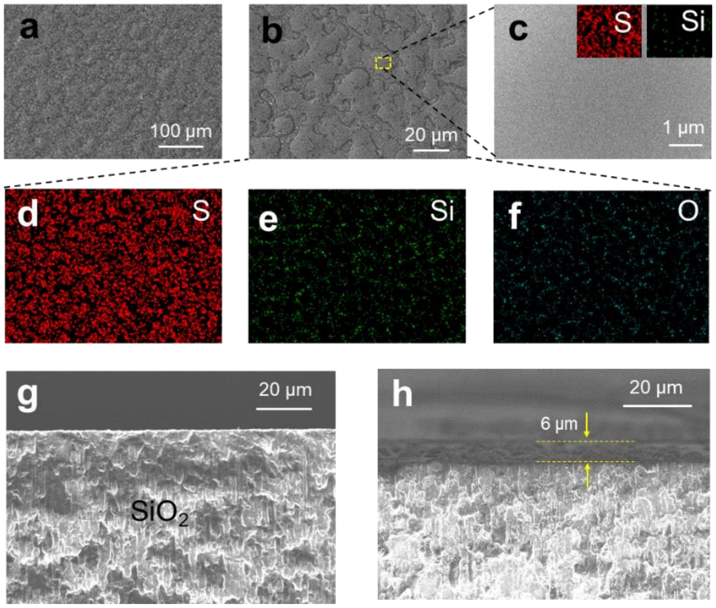

Supplementary Fig. 34 SEM and corresponding EDS mapping images of SiO_2 and SiO_2-S_d. a-c SEM images of the SiO_2-S_d surface at different scales. Insets in c: the corresponding EDS mapping images. **d-f** EDS mapping images corresponding to b. SEM images of the cross section of **g** SiO_2 and **h** SiO_2-S_d.

Supplementary Tables

Supplementary Table 1 The Hg^0 adsorption breakthrough ratio of different Al_2O_3@MS and Al_2O_3@MS-S_d.

Adsorbents	Breakthrough ratio (0~180 min)	Adsorbents	Breakthrough ratio (0~180 min)
Al_2O_3@CuS	8.1%~90.2%	Al_2O_3@CuS-S_d	3.0%~8.1%
Al_2O_3@ZnS	87.9%~99.4%	Al_2O_3@ZnS-S_d	20.6%~36.1%
Al_2O_3@CdS	79.0%~93.6%	Al_2O_3@CdS-S_d	9.9%~30.8%
Al_2O_3@In_2S_3	82.0%~93.6%	Al_2O_3@In_2S_3-S_d	25.0%~42.8%
Al_2O_3@PbS	58.6%~84.4%	Al_2O_3@PbS-S_d	23.1%~44.4%
Al_2O_3@SnS	69.1%~92.5%	Al_2O_3@SnS-S_d	39.1%~62.5%
Al_2O_3@Fe_2S_3	51.1%~93.5%	Al_2O_3@Fe_2S_3-S_d	66.3%~67.5%
Al_2O_3@CoS	48.6%~88.6%	Al_2O_3@CoS-S_d	65.5%~78.6%
Al_2O_3@MnS	70.0%~91.6%	Al_2O_3@MnS-S_d	55.9%~83.5%
Al_2O_3@NiS	81.9%~100%	Al_2O_3@NiS-S_d	71.7%~88.1%

* Reaction conditions: sorbent mass = 0.3 g, total flow rate = 360 mL min^{-1}, SO_2 concentration = 5000 ppm (during S-CVD process), H_2S concentration = 100 ppm (during S-CVD process), S-CVD time = 15 min, Hg^0 concentration = (1.5±0.05) mg m^{-3}, reaction temperature = 80 °C, and reaction time = 180 min.

Supplementary Table 2 Structural characterization of the crystalline form of different metal sulfides[16].

Material	Crystal system	Space group symbol	M-S bond length (Å)
CuS	Hexagonal	$P6_3/mmc$	2.18, 2.28, 2.35
ZnS	Cubic	$F\overline{4}\overline{3}m$	2.33
HgS	Cubic	$F\overline{4}\overline{3}m$	2.57
CdS	Cubic	$Fm\overline{3}m$	2.75
In_2S_3	Trigonal	$\overline{R}3c$	2.57, 2.71
PbS	Cubic	$Fm\overline{3}m$	2.98-3.00
SnS	Cubic	$Fm\overline{3}m$	2.91
Fe_2S_3	Trigonal	$\overline{R}3c$	2.22, 2.31
CoS	Hexagonal	$P6_3/mmc$	2.32
MnS	Cubic	$Fm\overline{3}m$	2.14
NiS	hexagonal	$P6_3/mmc$	2.37

Supplementary Table 3 The deposition rate of S_d^0 on Al_2O_3 and Al_2O_3@MS at different stages (mg $g^{-1}\cdot min^{-1}$).

Sorbent	0-5 min	5-30 min	>30 min
Al_2O_3	3.2	0.24	0.061
Al_2O_3@ZnS	2.5	0.23	0.062
Al_2O_3@PbS	1.8	0.22	0.052
Al_2O_3@CuS	2.0	0.15	0.046

Supplementary Table 4 Specific surface area, pore volume, average pore diameter of different materials.

Sorbent	Surface area (m^2 g^{-1})	Total pore volume (cm^3 g^{-1})	Average pore diameter (nm)
Al_2O_3	242.3	0.50	7.6
Al_2O_3@ZnS	259.0	0.45	6.4
Al_2O_3@ZnS-S_d*	244.4	0.42	6.3

* 30 min S-CVD on Al_2O_3@ZnS.

Supplementary Table 5 Surface sulfur valence analysis of different adsorbents.

Adsorbent	S^{2-}	S_n^{2-}	SO_4^{2-}
Al_2O_3@ZnS	45.0%	-	55.0%
Al_2O_3@ZnS-S_d	35.4%	36.7%	27.9%
Spent Al_2O_3@ZnS-S_d	82.9%	3.0%	14.1%
Al_2O_3@CuS	20.7%	21.6%	57.7%
Al_2O_3@CuS-S_d	19.1%	29.7%	51.2%
Al_2O_3@HgS	42.4%	-	57.6%
Al_2O_3@HgS-S_d	19.1%	35.4%	45.5%

Supplementary Table 6 Zn K-edge EXAFS fitting parameters of Zn foil, ZnO, ZnS, and ZnS-S_d.

Sample	Shell	CN[a]	R (Å)[b]	σ^2 (10^{-2}×Å^2)[c]	ΔE_0 (eV)[d]	R factor (Å)
Zn foil	Zn–Zn	6	2.66	1.24	2.2	0.0045
	Zn–Zn	6	2.84	3.07	4.5	
ZnO	Zn–O	4.0±0.5	1.96	0.36	3.6	0.0162
	Zn–Zn	12.1±2.1	3.24	1.54	3.9	
ZnS	Zn–S	3.9±0.2	2.33	0.61	3.6	0.0048
ZnS-S_d	Zn–S	3.5±0.3	2.43	0.58	3.1	0.0108

[a]*CN*, coordination number; [b]*R*, the distance to the neighboring atom; [c]σ^2, the mean square relative displacement (MSRD); [d]ΔE_0, inner potential correction; *R* factor indicates the goodness of the fit. S_0^2 = 0.865 (according to the experimental EXAFS fit of Zn foil by fixing *CN* = 6 as the known crystallographic value. Fitting range: 3.0 ≤ *k* (/Å) ≤ 10.0 and 1.0 ≤ *R* (Å) ≤ 3.0 (Zn foil); 2.5 ≤ *k* (Å^{-1}) ≤ 12.0 and 1.0 ≤ *R* (Å) ≤ 3.2 (ZnO); 3.0 ≤ *k* (Å^{-1}) ≤ 12.0 and 1.0 ≤ *R* (Å) ≤ 2.3 (ZnS); 3.0 ≤ *k* (Å^{-1}) ≤ 12.0 and 1.0 ≤ *R* (Å) ≤ 2.3 (ZnS-S_d). A reasonable range of EXAFS fitting parameters: 0.700 < S_0^2 < 1.000; *CN* > 0; σ^2 > 0 Å^2; |ΔE_0| < 15 eV; *R* factor < 0.02 Å.

Supplementary Table 7 Comparison of adsorption capacity of different kinds of mercury sorbents.

	Sorbents	Gas component	Adsorption capacity (mg/g)	Ref.
Carbon-based	ACN-AR	N_2	0.236 (100%)[a]	17
	Coal (FGD)	$6\%O_2+12\%CO_2+7\%H_2O$	0.573 (100%)	18
	Biomass (PAC)	$6\%O_2+12\%CO_2+7\%H_2O$	0.383 (100%)	
	Fly ash carbon	$16\%CO_2+5\%O_2+2000ppmSO_2$	1.85 [350 min][b]	19
	S/AC	N_2	2.3 (100%)	20
	Cu-BTC	$10\%O_2$(15 ppm HCl for 5 min)	1.7 [2 h]	21
Oxide-based	α-MnO_2	$4\%O_2$	~6.94 [10 h]	22
	15%-Mn/γ-Fe_2O_3-250	air	3.54 (55%)	23
	MnO_2/CS	N_2	~4.78 [10 h]	24
	$(Fe_2Ti)0.8O_4$	air	3.94 (23%)	25
	MnO_x/graphene-30%	4% O_2	2.7 [10 h]	26
	$Ce_{0.5}Mn_{0.5}O_y$	$4\%O_2$+500ppmNO+500ppm SO_2	5.6 [10 h]	27
	$(Fe_{2.2}Mn_{0.8})_{1-\delta}O_4$	$10\%O_2$	4.44 [10 h]	28
	Fe-Sn-MnO_x(1:20:20)	$4\%O_2$	~3.75 [10 h]	29
	Fe-Ti-Mn spinel	$4\%O_2$	2.3 [10 h]	30
	$LaMnO_3$	$8\%O_2$	6.8 [10 h]	31
	MnOx/CeO_2-TiO_2	400ppmNO+400ppmCO	9.4 (100%)	32
Sulfide-based	Nano-CuS	N_2	122.4 (100%)	33
	Fe_3O_4@CuS	N_2	88.7 (100%)	34
	in-situ etching ZnS	N_2	53.83 (50%)	7
	ex-situ etching ZnS	N_2	6.14 (50%)	7
	$FeS_{1.32}Se_{0.11}$	$6\%O_2+2.5\%SO_2+5\%H_2O$	20.216 (100%)	35
	$[MoS_4]^{2-}$/CoFe-LDH	$4\%O_2$	16.39 (100%)	36
	MoS_3/TiO_2	N_2	14.9 (75%)	37
	CoS_x	$4\%O_2$	43.03 (50%)	38
	CoMoS/γ-Al_2O_3	N_2	18.94 (100%)	39
	In_2S_3/g-C_3N_4	N_2	14.78 (100%)	40
	$CuInS_2$	N_2	13.81 (100%)	41
	40ZIS/CN	N_2	13.04 (100%)	42
	CuS/Al_2O_3	N_2	20.96 (100%)	43
	Mn-Sn_2S_6	N_2	21.05 (100%)	44
	Cu_2S	N_2	26.6 (100%)	45
	$CuFeS_2$	N_2	37.24 (100%)	46
	$FeMoS_x/TiO_2$	N_2	41.8 (100%)	47
	Co_9S_8	N_2	43.18 (100%)	48
	MoS_2	N_2	54.308 (100%)	49
	ZnO@CuS	N_2	60.53 (100%)	50
	Al_2O_3@ZnS-S_d	$5000ppmSO_2+4\%H_2O$ $+100ppmH_2S$(30min/24h)	303.9	This work

a: Adsorption breakthrough ratio; b: Adsorption time.

Supplementary Table 8 Summary of experimental conditions.

Experiment set	Sorbents	Weight (g)	Reaction temperature (°C)	Activation time[a]	Gas components	Total flow rate (mL min^{-1})
Set I	Al_2O_3 and Al_2O_3@MS[b]	0.3	80	0/15 min	1.5 mg m^{-3} Hg^0	360
Set II	Al_2O_3@ZnS	0.3	80	5/10/15/30/60 /240/480/1440 min	1.5 mg m^{-3} Hg^0	360
Set III	Al_2O_3@ZnS and Al_2O_3@CuS	0.3	60/80/100/ 120/140/160	30 min	1.5 mg m^{-3} Hg^0	360
Set IV	Al_2O_3@ZnS	0.3	120	30 min	1.5 mg m^{-3} Hg^0,5000 ppm SO_2/ 5% O_2/100 ppm NO/4% H_2O	360
Set V	Al_2O_3@ZnS	0.3	120	30 min	1.5 mg m^{-3} Hg^0, 0/5000/33000 ppm SO_2	360
Set VI	Al_2O_3@ZnS, Al_2O_3@CuS and Al_2O_3@HgS	0.4	60/120	30 min per 24 h	2.5 mg m^{-3} Hg^0, 5000 ppm SO_2, 4% H_2O	300
Set VII	Natural chalcophile ore<br>Natural sphalerite ore	1.0	40<br>120	30 min per 24 h<br>30 min per 12/6 h	2.5 mg m^{-3} Hg^0, 6% SO_2, 4% H_2O	300

a. Activation condition: 100 ppm H_2S, 5000 ppm SO_2 (for set I, II, III, and V), activation temperature = reaction temperature.
b. M = Cu, Zn, Hg, In, Fe, Cd, Pb, Co, Ni, and Mn.

Supplementary References

1 Ravel, B. & Newville, M. ATHENA, ARTEMIS, HEPHAESTUS: data analysis for X-ray absorption spectroscopy using IFEFFIT. *Journal of Synchrotron Radiation* **12**, 537-541 (2005).

2 Azizian, S. Kinetic models of sorption: a theoretical analysis. *Journal of Colloid and Interface Science* **276**, 47-52 (2004).

3 Ho, Y. S. & McKay, G. Pseudo-second order model for sorption processes. *Process Biochemistry* **34**, 451-465 (1999).

4 Mi, X. *et al.* Preparation of graphene oxide aerogel and its adsorption for Cu^{2+} ions. *Carbon* **50**, 4856-4864 (2012).

5 Ho, Y. S. & McKay, G. A Comparison of Chemisorption Kinetic Models Applied to Pollutant Removal on Various Sorbents. *Process Safety and Environmental Protection* **76**, 332-340 (1998).

6 Perdew, J. P., Burke, K. & Ernzerhof, M. Generalized gradient approximation made simple. *Phys. Rev. Lett.* **78**, 1396-1396 (1997).

7 Li, H. *et al.* In situ acid etching boosts mercury accommodation capacities of transition metal sulfides. *Nat. commun.* **14**, 1395 (2023).

8 Li, Y. *et al.* Photoreduction of inorganic carbon (+IV) by elemental sulfur: Implications for prebiotic synthesis in terrestrial hot springs. *Sci. Adv.* **6**, eabc3687 (2020).

9 Yang, Y. *et al.* Different Crystal Forms of ZnS Nanomaterials for the Adsorption of Elemental Mercury. *Environ. Sci. Technol.* **55**, 6965-6974 (2021).

10 Xu, W. *et al.* Fundamental mechanistic insights into the catalytic reactions of Li─S redox by Co single-atom electrocatalysts via operando methods. *Sci. Adv.* **9**, eadi5108 (2023).

11 Li, W. *et al.* A high performance sulfur-doped disordered carbon anode for sodium ion batteries. *Energy & Environmental Science* **8**, 2916-2921 (2015).

12 Zeng, S. *et al.* Conducting Polymers Crosslinked with Sulfur as Cathode Materials for High-Rate, Ultralong-Life Lithium–Sulfur Batteries. *ChemSusChem* **10**, 3378-3386 (2017).

13 Penczek, S., Ślazak, R. & Duda, A. Anionic copolymerisation of elemental sulphur. *Nature* **273**, 738-739 (1978).

14 Baysal, M., Yürüm, A., Yıldız, B. & Yürüm, Y. Structure of some western Anatolia coals investigated by FTIR, Raman, 13C solid state NMR spectroscopy and X-ray diffraction. *Int. J. Coal Geol.* **163**, 166-176 (2016).

15 Ovchinnikov, O. V. *et al.* Manifestation of intermolecular interactions in FTIR spectra of methylene blue molecules. *Vib. Spectrosc* **86**, 181-189 (2016).

16 Jain, A. *et al.* Commentary: The Materials Project: A materials genome approach to accelerating materials innovation. *APL Mater.* **1**, 011002

(2013).
17 Li, Y., Lee, C. & Gullett, B. The effect of activated carbon surface moisture on low temperature mercury adsorption. *Carbon* **40**, 65-72 (2002).
18 Hsi, H. C., Rood, M. J., Rostam-Abadi, M., Chen, S. & Chang, R. Mercury adsorption properties of sulfur-impregnated adsorbents. *J. Environ, Eng.* **128**, 1080-1089 (2002).
19 Maroto-Valer, M. M., Zhang, Y. Z., Granite, E. J., Tang, Z. & Pennline, H. W. Effect of porous structure and surface functionality on the mercury capacity of a fly ash carbon and its activated sample. *Fuel* **84**, 105-108 (2005).
20 Liu, W., Vidic, R. D. & Brown, T. D. Optimization of sulfur impregnation protocol for fixed bed application of activated carbon-based sorbents for gas-phase mercury removal. *Environ. Sci. Technol.* **32**, 531-538 (1998).
21 Chen, D., Zhao, S., Qu, Z. & Yan, N. Cu-BTC as a novel material for elemental mercury removal from sintering gas. *Fuel* **217**, 297-305 (2018).
22 Xu, H. *et al.* Different crystal-forms of one-dimensional MnO_2 nanomaterials for the catalytic oxidation and adsorption of elemental mercury. *J. Hazard. Mater.* **299**, 86-93 (2015).
23 Yang, S. *et al.* Capture of gaseous elemental mercury from flue gas using a magnetic and sulfur poisoning resistant sorbent Mn/gamma-Fe_2O_3 at lower temperatures. *J. Hazard. Mater.* **186**, 508-515 (2011).
24 Xu, H. *et al.* Design of 3D MnO_2/Carbon Sphere Composite for the Catalytic Oxidation and Adsorption of Elemental Mercury. *J. Hazard. Mater.* **342**, 69-76 (2017).
25 Yang, S. *et al.* Nanosized cation-deficient Fe−Ti spinel: a novel magnetic sorbent for elemental mercury capture from flue gas. *ACS Appl. Mater. Inter.* **3**, 209-217 (2011).
26 Xu, H. *et al.* MnO_x/Graphene for the Catalytic Oxidation and Adsorption of Elemental Mercury. *Environ. Sci. Technol.* **49**, 6823-6830 (2015).
27 Qu, Z., Xie, J., Xu, H., Chen, W. & Yan, N. Regenerable Sorbent with a High Capacity for Elemental Mercury Removal and Recycling from the Simulated Flue Gas at a Low Temperature. *Energ. Fuel.* **29**, 6187-6196 (2015).
28 Yang, S. *et al.* Gaseous elemental mercury capture from flue gas using magnetic nanosized $(Fe_{3-x}Mn_x)_{1-\delta}O_4$. *Environ. Sci. Technol.* **45**, 1540-1546 (2011).
29 Xu, H. *et al.* The cooperation of Fe-Sn in a MnO_x complex sorbent used for capturing elemental mercury. *Fuel* **140**, 803-809 (2015).
30 Liao, Y. *et al.* The Centralized Control of Elemental Mercury Emission from the Flue Gas by a Magnetic Rengenerable Fe-Ti-Mn Spinel. *J. Hazard. Mater.* **299**, 740-746 (2015).
31 Xu, H. *et al.* Catalytic oxidation and adsorption of Hg^0 over low-temperature NH_3-SCR $LaMnO_3$ perovskite oxide from flue gas. *Appl.*

Catal. B-Environ. **186**, 30-40 (2016).

32 He, J., Reddy, G. K., Thiel, S. W., Smirniotis, P. G. & Pinto, N. G. Simultaneous Removal of Elemental Mercury and NO from Flue Gas Using CeO2 Modified MnOx/TiO2 Materials. *Energ. Fuel.* **27**, 4832-4839 (2013).

33 Yang, Z. *et al.* Multiform Sulfur Adsorption Centers and Copper-Terminated Active Sites of Nano-CuS for Efficient Elemental Mercury Capture from Coal Combustion Flue Gas. *Langmuir* **34**, 8739-8749 (2018).

34 Yang, Z. *et al.* Magnetic Rattle-Type Fe_3O_4@CuS Nanoparticles as Recyclable Sorbents for Mercury Capture from Coal Combustion Flue Gas. *ACS Appl. Nano Mater.* **1**, 4726-4736 (2018).

35 Liu, Z. *et al.* Development of Recyclable Iron Sulfide/Selenide Microparticles with High Performance for Elemental Mercury Capture from Smelting Flue Gas over a Wide Temperature Range. *Environ. Sci. Technol.* **54**, 604-612 (2020).

36 Xu, H. M. *et al.* $[MoS_4]^{2-}$ Cluster Bridges in Co-Fe Layered Double Hydroxides for Mercury Uptake from S-Hg Mixed Flue Gas. *Environ. Sci. Technol.* **51**, 10109-10116 (2017).

37 Mei, J. *et al.* Outstanding Performance of Recyclable Amorphous MoS_3 Supported on TiO_2 for Capturing High Concentrations of Gaseous Elemental Mercury: Mechanism, Kinetics, and Application. *Environ. Sci. Technol.* **53**, 4480-4489 (2019).

38 Quan, Z. *et al.* Study on the regenerable sulfur-resistant sorbent for mercury removal from nonferrous metal smelting flue gas. *Fuel* **241**, 451-458 (2019).

39 Zhao, H. *et al.* Hg^0 Capture over CoMoS/gamma-Al_2O_3 with MoS_2 Nanosheets at Low Temperatures. *Environ. Sci. Technol.* **50**, 1056-1064 (2016).

40 Zhou, M. *et al.* Spherical In_2S_3 anchored on g-C_3N_4 nanosheets for efficient elemental mercury removal in the wide temperature range. *Chem. Eng. J.* **430**, 132857 (2022).

41 Yu, Y., Yang, Y., Liu, J., Ding, J. & Zhang, J. Nanosized Cu-In spinel-type sulfides as efficient sorbents for elemental mercury removal from flue gas. *Sci. Total Environ.* **796**, 149094 (2021).

42 Wang, F. *et al.* Bimetallic sulfides $ZnIn_2S_4$ modified g-C_3N_4 adsorbent with wide temperature range for rapid elemental mercury uptake from coal-fired flue gas. *Chem. Eng. J.* **426**, 131343 (2021).

43 Hong, Q. *et al.* Adsorption of Gaseous Mercury for Engineering Optimization: From Macrodynamics to Adsorption Kinetics and Thermodynamics. *ACS EST Engg.* **1**, 865-873 (2021).

44 Hong, Q. *et al.* Regulation of the Sulfur Environment in Clusters to Construct a Mn–Sn2S6 Framework for Mercury Bonding. *Environ. Sci. Technol.* **56**, 2689–2698 (2022).

45 Wang, L. *et al.* Fabrication of Cu_2S hollow nanocages with enhanced high-temperature adsorption activity and recyclability for elemental mercury capture. *Chem. Eng. J.* **427**, 130935 (2022).
46 Yang, J. *et al.* Recyclable chalcopyrite sorbent for mercury removal from coal combustion flue gas. *Fuel* **290**, 120049 (2021).
47 Wang, C., Lv, P., Ma, Y., Mei, J. & Yang, S. Simultaneous Adsorption of Gaseous Hg^0 and Hg(II) by Regenerable Monolithic $FeMoS_x/TiO_2$: Mechanism and its Application in the Centralized Control of Hg Pollution in Coal-Fired Flue Gas. *Environ. Sci. Technol.* **56**, 10977-10986 (2022).
48 Yang, S. *et al.* Co_9S_8 nanoparticles-embedded porous carbon: A highly efficient sorbent for mercury capture from nonferrous smelting flue gas. *J. Hazard. Mater.* **412**, 124970 (2021).
49 Xiao, Y. *et al.* Coordinative sulfur site over flower-structured MoS_2 for efficient elemental mercury uptake from coal-fired flue gas. *Chem. Eng. J.* **434**, 134649 (2022).
50 Hong, Q. *et al.* Shell-thickness-induced spontaneous inward migration of mercury in porous ZnO@CuS for gaseous mercury immobilization. *Chem. Eng. J.* **420**, 127592 (2021).
